# Experimental Models of Hepatocellular Carcinoma—A Preclinical Perspective

**DOI:** 10.3390/cancers13153651

**Published:** 2021-07-21

**Authors:** Alexandru Blidisel, Iasmina Marcovici, Dorina Coricovac, Florin Hut, Cristina Adriana Dehelean, Octavian Marius Cretu

**Affiliations:** 1 Faculty of Medicine, “Victor Babeș” University of Medicine and Pharmacy Timisoara, Eftimie Murgu Square No. 2, RO-300041 Timișoara, Romania; blidy@umft.ro (A.B.); florin.hut@umft.ro (F.H.); octavian.cretu@umft.ro (O.M.C.); 2 Faculty of Pharmacy, “Victor Babeș” University of Medicine and Pharmacy Timisoara, Eftimie Murgu Square No. 2, RO-300041 Timișoara, Romania; iasmina.marcovici@umft.ro; 3 Research Center for Pharmaco-Toxicological Evaluations, Faculty of Pharmacy, “Victor Babes” University of Medicine and Pharmacy Timisoara, Eftimie Murgu Square No. 2, RO-300041 Timișoara, Romania

**Keywords:** hepatocellular carcinoma, 2D cell lines, 3D tumor spheroids, organoids, organ-on-a-chip, mouse models, in silico, machine learning, artificial intelligence algorithms

## Abstract

**Simple Summary:**

Hepatocellular carcinoma (HCC) is characterized by a broad molecular and genetic heterogeneity, which makes it a challenging subject in terms of the underlying mechanisms, response and resistance to treatment, and finding novel therapeutic options. Nowadays, new experimental models (3D in vitro models, in vivo mouse and non-mouse models, and computational studies) allow more detailed studies of hepatocellular carcinoma pathogenesis and treatment. Here, we provide insights into the current preclinical models frequently applied for the study of hepatocellular carcinoma.

**Abstract:**

Hepatocellular carcinoma (HCC), the most frequent form of primary liver carcinoma, is a heterogenous and complex tumor type with increased incidence, poor prognosis, and high mortality. The actual therapeutic arsenal is narrow and poorly effective, rendering this disease a global health concern. Although considerable progress has been made in terms of understanding the pathogenesis, molecular mechanisms, genetics, and therapeutical approaches, several facets of human HCC remain undiscovered. A valuable and prompt approach to acquire further knowledge about the unrevealed aspects of HCC and novel therapeutic candidates is represented by the application of experimental models. Experimental models (in vivo and in vitro 2D and 3D models) are considered reliable tools to gather data for clinical usability. This review offers an overview of the currently available preclinical models frequently applied for the study of hepatocellular carcinoma in terms of initiation, development, and progression, as well as for the discovery of efficient treatments, highlighting the advantages and the limitations of each model. Furthermore, we also focus on the role played by computational studies (in silico models and artificial intelligence-based prediction models) as promising novel tools in liver cancer research.

## 1. Introduction

Hepatocellular carcinoma (HCC), the most common form of hepatic malignancy arising due to the accumulation of genomic and epigenomic alterations in hepatocytes [1,2], is ranked among the deadliest types of cancer worldwide [3]. Its features include massive molecular heterogeneity [1], poor prognosis, strong metastatic capacity, high frequency of recurrence [3], and lack of effective therapeutic strategies [4]. Its incidence is expected to increase in the future. HCC generally develops within the setting of a pre-existing liver condition (e.g., chronic inflammation, fibrosis, cirrhosis, fatty liver disease), being almost exclusively caused by infectious factors, including HBV (Hepatitis B virus) and HCV (Hepatitis C virus) infection, by environmental factors such as exposure to carcinogens (e.g., aflatoxin B), and chronic alcohol consumption. The broad spectrum of etiologies is reflected in the molecular heterogeneity of HCC [1,5,6,7].

The treatment strategies for HCC vary among patients and are highly dependent on the disease stage [8]. Early HCC stages are currently curable by complete surgical resection or liver transplantation. In intermediate stages, HCC patients may benefit from locoregional therapies, including transarterial chemoembolization, ablation, and selective internal radiotherapy [9]. However, the treatment options for patients with advanced HCC are systemic therapies such as chemo- and immunotherapy [8,10], which are limited and poorly efficient [3] due to several factors such as late diagnosis (approximately 85% of HCC patients), underlying liver damage [8], and tumor resistance to chemotherapy [11]. Therefore, the development of novel curative strategies is crucial. To achieve this goal, gathering a mechanistic understanding of HCC pathogenesis is essential. Over the last few decades, substantial progress has been made in the establishment of highly accurate cancer systems [12], a wide range of HCC models being available. In particular, preclinical (in vitro and in vivo) platforms simulating the major characteristics of human HCC have emerged as essential tools for understanding the tumor biology and establishing proper therapeutic candidates [3,13].

Driven by the recent progress recorded in cancer research, in this review, we offer a thorough discussion regarding the currently available preclinical models frequently applied in the pathological and therapeutic investigations of HCC, highlighting the advantages and the limitations of each model. In addition, the role played by computational studies (in silico models and artificial intelligence-based prediction models) as promising novel tools in liver cancer research is also discussed.

## 2. General Aspects of HCC

HCC is the most habitual malignancy that arises due to the malignant transformation of hepatocytes, accounting for 90% of all primary liver cancer diagnostics [14]. HCC remains a major health concern worldwide [14], with an incidence that is expected to rise in the future [1]. Plenty of etiological risk factors have been strongly associated with HCC development. Above all, hepatotropic viruses such as HBV and HCV are leading causes in the occurrence of liver cancer, the global distribution of HCC reflecting the rate of these viral infections [15]. Thus, the greatest HCC prevalence is in the Asian regions and sub-Saharan Africa due to the endemic HBV infection. In contrast, HCV infection and nonalcoholic fatty liver disease (NAFLD) are major risk factors associated with HCC in western countries [1,16]. Another infectious factor considered to augment the risk of developing HCC is represented by the Hepatitis D–delta virus (HDV), a hybrid virus that acts as a satellite of HBV by incorporating the HBV surface antigen (HBsAg) and infecting only the persons that present an infection with HBV [17]. In addition, exposure to the Aspergillus-derived aflatoxin, obesity, smoking, oral contraception use, and alcohol intake are primary risk factors contributing to liver carcinogenesis [15,18]. Generally, HCC is preceded by a chronic liver illness, such as cirrhosis, fibrosis, or fatty liver disease [7,14]. The unremitting cirrhosis is considered a precancerous environment due to the immune responses’ imbalance that lead to this disorder (as chronic inflammation and destruction of hepatocytes), while the inflammatory background, the reactive oxygen species (ROS) generation, and the genomic DNA mutations represent dominant factors in augmenting the malignant transformation of hepatocytes [19]. The dysregulation of immune mechanisms observed in HCC highlights the immunogenic features of this type of cancer, a subject that is still underexplored [19,20].

**Figure 1:**
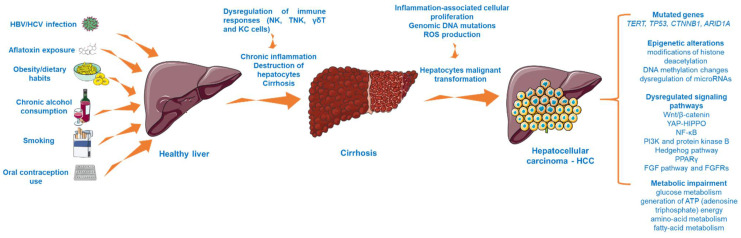
The multistep process involved in HCC development. This image contains Servier Medical Art elements, which are licensed under a Creative Commons Attribution 3.0 Unported License; https://smart.servier.com, accessed on 20 June 2021. The following abbreviations are used in the figure: HBV—hepatitis B virus; HCV—hepatitis C virus; NK—natural killer cells; TNK—natural killer T cells; KC—Kupffer cells; ROS—reactive oxygen species; TERT—telomerase reverse transcriptase; TP53—tumor protein 53; YAP-HIPPO—Yes-Associated Protein-Hippo Pathway, PPARγ—peroxisome proliferator-activated receptor gamma; FGF—fibroblast growth factor.

HCC is considered a multistep process (Figure 1) arising from the malignant transformation of hepatocytes that acquire different genomic and epigenomic changes [21] and is characterized by high heterogeneity, both from a clinical and molecular perspective [22]. Despite the fact that the precise molecular mechanisms that trigger HCC initiation and progression are far from being completely elucidated, recent research has revealed the landscape of mutated genes and dysregulated signaling pathways associated with HCC [23]. For instance, abundant evidence has indicated the importance of telomere maintenance in hepatocarcinogenesis [24], the TERT (telomerase reverse transcriptase) promoter mutations being the main somatic alteration observed in HCC samples [22]. In addition, the oncogenes and tumor suppressor genes TP53, CTNNB1 (catenin beta-1), and ARID1A (AT-rich interactive domain-containing protein 1A) are recurrently mutated genes in progressed HCC [23,24]. Another frequent abnormality found in HCC is the mutation of the tumor protein 53 (p. 53) [25]. Several signaling pathways (e.g., Wnt/β-catenin, phosphatidylinositol-3-kinase and protein kinase B, Hedgehog pathway, Yes-Associated Protein-Hippo Pathway (YAP-HIPPO), NF-κB pathway, regulation of lipid metabolism by peroxisome proliferator-activated receptor gamma (PPARγ), and fibroblast growth factor (FGF) pathway and its receptors (FGFRs)) have been identified as vital to HCC occurrence [26,27,28]. In particular, the overactivation of the Wnt/β-catenin pathway, which regulates cancer cell proliferation, invasion, survival, and apoptosis, is frequently found in HCC [26]. Another mechanism considered to be involved in the development of hepatocarcinogenesis is represented by epigenetic alterations, such as modifications of histone deacetylation (histone deacetylases 3 and 6, and histone 3 lysine 9 acetylation (H3K9)), DNA methylation changes (overexpression of DNA methyltransferase 1 (DNMT1), hypermethylation of p16, p15, and E-cadherin genes), and dysregulation of microRNAs (miR-122—low expression and miR-221—overexpression) [27]. Besides the genetic and epigenetic abnormalities specific for HCC, there were also noticed alterations of several metabolic processes such as glucose metabolism, generation of ATP (adenosine triphosphate) energy, and amino acid and fatty acid metabolism, changes that increase the capacity of tumor cells to expand, proliferate, and metastasize [29].

The aggressiveness of HCC cells has been correlated to their ability to interact with stromal cells and the extracellular environment [30], which has been shown to play a vital role in HCC initiation, progression, metastasis, and response to treatment. Additionally, the tumor heterogeneity is highly dependent on the microenvironment composition, which generally includes a cellular component (HCC cells, hepatic stellate cells, tumor-associated fibroblasts, immune system cells, etc.) and a non-cellular component (e.g., extracellular matrix, bioactive molecules) [31].

Serologic testing, computed tomography (CT) or magnetic resonance imaging (MRI), and histology exams are common tools in diagnosing HCC [14]. However, when diagnosed, the majority of HCC cases cannot benefit from curative interventions [32]. In early stages, the main HCC treatment strategies include surgical resection and liver transplantation [14], while in advanced forms, chemotherapy remains the mainstay of HCC treatment. To date, tyrosine kinase inhibitors (TKIs) represent the first-line systemic treatment for HCC management [10]. Notably, sorafenib was the first drug able to improve the overall survival rate in advanced forms of HCC [9], thus being the only TKI recommended in the treatment of this malignancy for over a decade [33]. Since then, other TKIs have been discovered, of which only lenvatinib has been proven as non-inferior to sorafenib during clinical trials [9]. Advanced HCC is recognized as refractory to systemic anticancer medication, which explains the lack of chemotherapeutic options [14].

## 3. Preclinical Experimental Models for HCC 

**Figure 2:**
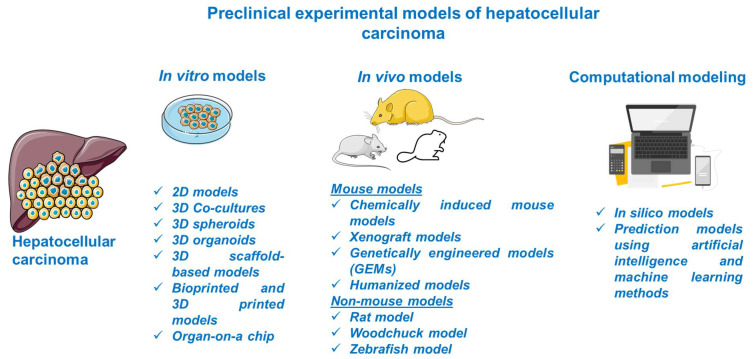
Schematic overview of the preclinical models of HCC discussed in the present study. This image contains Servier Medical Art elements, which are licensed under a Creative Commons Attribution 3.0 Unported License; https://smart.servier.com accessed on 20 June 2021.

Considerable progress was recorded in the field of HCC as regards its biology, pathogenesis, molecular, genetic, and epigenetic underlying mechanisms, and therapeutic innovations; the understanding of this complex process was obtained with the assistance of preclinical experimental models. The preclinical models offer relevant insights regarding the main features of human HCC, but they have several limitations since there is no model able to mimic and reproduce the integral heterogeneity and complexity of this type of cancer [3]. In light of these data, the selection of an appropriate model for research directions in the field of HCC represents a crucial step and involves a thorough documentation. This review describes the main in vitro, in vivo, and computational methods for modeling HCC (Figure 2).

### 3.1. In Vitro HCC Models 

**Figure 3:**
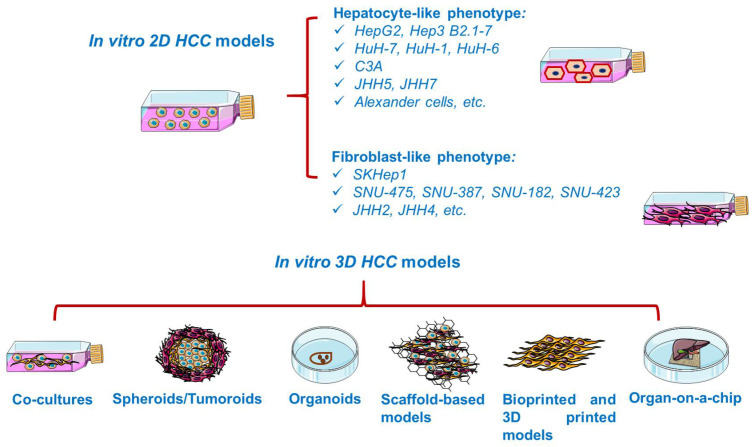
Overview of the in vitro established models for HCC. This image contains Servier Medical Art elements, which are licensed under a Creative Commons Attribution 3.0 Unported License; https://smart.servier.com accessed on 20 June 2021.

In vitro studies are generally based on the growth of isolated cells in specific culture media [34]. In vitro cancer models (Figure 3) vary in complexity from simplistic two-dimensional (2D) monolayers to advanced three-dimensional (3D) models resembling the tumor microenvironment [35]. Cancer cell lines are valuable preclinical models routinely used in cancer research and drug discovery [36], providing insights into cellular signaling pathways, metabolism, invasion, proliferation [34], and response to chemotherapy or radiotherapy, drug resistance [37], as well as the molecular mechanisms of tumor growth and metastasis [35]. However, 2D cell lines display several shortcomings, such as cross-contamination with other lines and proneness to genetic drift after long-term culturing [38], deviating considerably the in vivo response [39]. Moreover, 2D cultured cells are constrained to adhere to a rigid surface, adopting a flat morphology, which affects the normal cellular functions such as signaling, proliferation, migration, and apoptosis [37,40]. In addition, under 2D conditions, the extracellular matrix (ECM) constituents and cell–cell and cell–ECM interactions are negatively impacted [37]. Despite their inability to replicate the complicated tumor environment, 2D cell cultures remain the foundation of today’s cancer drug discovery, current preclinical research relying heavily on cells grown in monolayers [38].

Three-dimensional (3D) models are innovative instruments for in vitro cancer research, developed from either commercialized cell lines or patient-derived biopsies [34], which have become widely popular in modern drug screening and tissue engineering [41]. As compared to conventional cell cultures, 3D systems provide a much more realistic preservation of the in vivo conditions, processes [42,43], and microenvironment where the tumor resides and develops [44]. The 3D platforms fill the gap between 2D cultures and animal testing, improving the success rate of novel anticancer drug discovery [41]. In addition, they permit the evaluation of several cellular aspects such as proliferation, morphology, motility, adhesion, and signaling, as well as the analysis of cell–cell and cell–microenvironment interplay [45]. Nonetheless, several limitations have been described, such as laborious processing, reduced application in high-throughput screening, a limited survival period in culture, and the paucity of reliable experimental protocols [46].

#### 3.1.1. 2D HCC Models

Cell lines, both of human and animal origin, have been extensively applied in the modeling of HCC since they carry the majority of genetic and epigenetic alterations present in the tumor of origin [3,13]. However, it is a matter of debate how well these cell lines preserve the biological features of primary HCC and its response to treatment [13]. Major applications of HCC cell lines include the study of cell proliferation and metastatic progression. Furthermore, HCC cell lines are broadly used as tools for molecular target discovery, drug screening, and in vivo xenograft development [3], allowing rapid and cost-efficient evaluation of potential therapeutics [40]. Thus far, HCC 2D systems are limited to around 30 publicly available cell lines [47]. A very recent study investigated the gene expression profile of 28 liver carcinoma cell lines, as a relevant criterion for selecting a suitable cell line for HCC studies [48].

**Figure 4:**
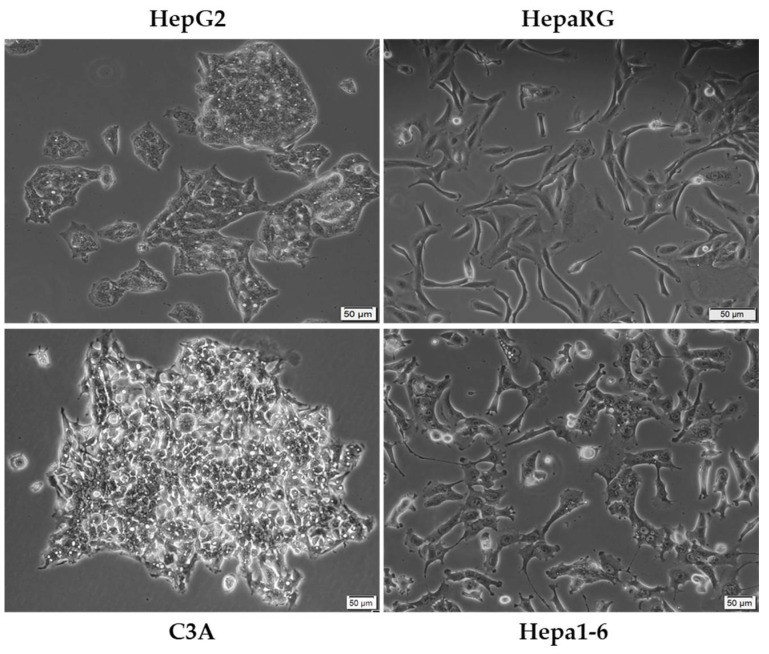
Morphological aspect of some of the most frequently used cell lines as in vitro models for HCC of human (HepG2, HepaRG, and C3A) and murine (Hepa1-6) origin. The scale bar represents 50 µM.

The most commonly employed experimental model for in vitro liver cancer research is the HepG2 (Figure 4) cell line due to its availability and complex characterization [49], being applied in toxicological and pharmacological investigations since the 1970s [50]. Deriving from the liver biopsy of a Caucasian adolescent, HepG2 is recognized as a cell line negative for hepatitis virus that exhibits intact features of human neoplastic lesions (e.g., increased expression of α-fetoprotein, α2-macroglobulin, and transferrin) [3]. In addition, the cells express many differentiated hepatic functions (e.g., synthesis of plasma proteins, bile acids, and glycogen, metabolization of cholesterol and triglycerides) [51]. Nevertheless, the use of the HepG2 cell line remains rather controversial, with several studies suggesting its non-tumorigenicity and descendance from an epithelial hepatoblastoma-like tumor [3,49]. C3A (Figure 4) is a subclone of the HCC HepG2 cell line [52], presenting a more hepatocyte-like morphology as compared to the ancestral line [53]. HepaRG cells (Figure 4) originate from a hepatic progenitor cell line of a female hepatocarcinoma associated with chronic hepatitis C and cirrhosis [50,54]. The cells exhibit a typical epithelial aspect, being able to differentiate in two phenotypically distinct cell populations: (i) large polygonal biliary-like cells with clear cytosol and refringent edges, and (ii) small hepatocyte-like cells with dark cytosol and prominent nuclei. The HepaRG cell line is a valuable tool in drug screening, drug metabolism studies, carcinogenesis, and HBV infection [54]. In stark comparison to the human cells presented so far, Hepa1-6 (Figure 4) is a murine hepatic cancer cell line originating from the spontaneous BW7756 tumor that developed in C57BL/J mice [55].

Other cell lines frequently used in liver cancer research and the differences established so far in the literature between them are summarized in Table 1. To verify the relevance of the selected cell lines in the preclinical evaluation of HCC, we performed a PubMed database search. According to the results, HepG2 is by far the most employed cell line in HCC research, followed by Hep3B, HuH-7, and C3A. The response to the HCC chemotherapeutic agent sorafenib varies among cell lines, Hep3B being the most sensitive, with a calculated IC_50_ value of 3.31 µM.

#### 3.1.2. 3D HCC Models

##### HCC Co-Cultures 

The co-culture technique enables the cultivation of a variety of cell types together in one culture dish, allowing the examination of cell–cell interactions, which have been reported as key players in cancer invasion [69,70]. Iwahashi and colleagues remarked that co-culturing HCC cells (HepG2 and HuH-7) with hepatic stellate cells (LX2 and Li90) promotes cancer progression via the IL-6/STAT3 pathway, increases the migratory potential of the cancer cells, and elevates the expression of the epithelial–mesenchymal transition marker E-cadherin and stem cell markers EpCAM and CD44 [71]. Additionally, co-cultures offer a better perspective on the response of HCC cells to therapy by mimicking the tumor microenvironment. In a study conducted by Chen et al., it was revealed that co-culturing LX2 hepatic stellate cells with HuH-7 HCC cells reversed the sorafenib-induced effects in the HuH-7 cell line, such as suppressed cell viability, increased PARP, and decreased levels of antiapoptotic proteins Mcl-1 and Bcl-2, enabling drug resistance via the HGF/c-Met/Akt and Jak2/Stat3 pathways [72] (Table 2).

##### HCC Spheroids

Tumor spheroids can be defined as sphere-shaped 3D cell aggregates [83,84], consisting of a necrotic core covered by layers of active and proliferating cells [34]. Spheroid cultures compensate for the deficiencies of 2D cultures [85] by retaining the in vivo characteristics in terms of morphology, phenotype, microenvironment, and cell–cell and cell–extracellular matrix communication [83], which makes them suitable for HCC studies [11]. Co-culturing normal and malignant cells within a spheroid can extend the knowledge about angiogenesis and tumor metastasis [84]. Among the widely employed applications of 3D spheroids are included the screening of anticancer therapy efficacy [86] and the study of responses to existing treatment interventions such as radiation, chemotherapy, and immunotherapy, or combined therapies [84].

Several spheroid-type cultures for the study of HCC have been described so far. For instance, Song and colleagues developed patient-derived multicellular tumor spheroids (MCTS) as a screening tool for personalized cancer therapy in HCC. In addition, the chemosensitivity of the system to sorafenib, 5-fluorouracil, and cisplatin has been assessed in comparison to monolayer cell cultures and classic tumor spheroids. MCTS exhibited a selective response to anticancer drugs as compared to homogeneous HCC spheroids [87]. Khawar et al. designed a stroma-rich mixed-cell spheroid model by co-culturing HuH-7 cells and LX2 stellate cells sharing several similarities with the in vivo tumor phenotype, such as a profibrotic microenvironment, chemotherapy resistance, and invasive cell movement [11]. However, taking into account the benefits that spheroids bring to the study of cancer, it is important to mention that they are not ideal platforms, mainly due to several inconsistencies in their formation, their challenging manipulation, and the absence of specific extracellular matrix constituents [88]. A summary characterization of all types of 3D in vitro models described in the present manuscript is presented in Table 3.

##### HCC Organoids

Organoids are 3D systems able to preserve the identity of the modeled organ, maintaining some of its physiological aspects and properties, including self-organization, self-renewal, multilineage differentiation, and histological features. Organoids provide several advantages. They enable long-term culturing, cryopreservation, and genetical manipulation similarly to conventional 2D platforms, combining the tractability of in vitro cell cultures with the architecture and differentiation of in vivo models [100]. Cancer organoid models permit the study of carcinogenesis, cancer metastasis, and drug screening for the discovery of personalized anticancer treatments [12].

Liver organoids are particularly interesting due to the numerous functions exerted by the human liver and their capacity to shape several liver diseases [101]. HCC organoids recapitulate the morphological aspects, as well as the expression of specific HCC tumor markers, preserving the genetic heterogeneity of the tumor from which they originated [102]. However, at present, these 3D systems exhibit several weaknesses, such as high costs, time-consuming and difficult preparation, and the lack of blood vessels or the immune components that play crucial roles in in vivo systems [3].

Nuciforo et al. developed tumor organoids from biopsies obtained from HCC patients that retained the histological and genomic characteristics of the parental tumors, presented variable sensitivity to chemotherapy (sorafenib), and generated tumors that also recapitulated the features of the original biopsy in immunodeficient mice [102]. Other examples of liver tumor organoids are presented in Table 2.

##### Scaffold-Based HCC Models

Another valuable 3D culture method is represented by scaffold-based platforms [37]. Scaffold-based models differ from the other 3D culture methods by providing a physical matrix on which cells are able to aggregate, divide, and migrate [96].

Scaffolds represent pre-fabricated 3D structures [96] that are made of a series of materials with different porosities, permeability, surface nature, and mechanical stability and are modulated in order to reconstruct the microenvironment or the extracellular matrix of specific tissues and tumors. In stark contrast to scaffold-free models, in scaffold-based 3D cultures, cells are embedded into the matrix, the physicochemical properties of the scaffold material strongly influencing cell characteristics [96]. In addition, this architecture enhances the interactions between the adhered cells, providing a support for them to proliferate and auto-organize [37]. Scaffolds can be natural or synthetic, specifically engineered to imitate the properties of the extracellular matrix. Several bioactive molecules such as growth factors and hormones can be added in synthetic scaffolds to increase cell proliferation or to induce a characteristic cell phenotype [96]. Beyond the positive features of these 3D systems, when selecting scaffold-based 3D culture platforms, several factors should be taken into consideration, factors that could be considered drawbacks of the model, including the composition heterogeneity of the materials between batches, thorough verification of materials’ properties (e.g., mechanical properties, swelling capacity, degradation) for each experiment, degradation products that could determine immunogenic reactions, reduced reproducibility of the results due to the variability in properties, and low mimicry of the human tumor environment [103].

Leung et al. developed a chitosan–alginate scaffold 3D system of HCC which closely imitates the in vivo tumor behavior and might serve as a proper model for the study of HCC therapeutic options. Their study revealed that, when grown on a chitosan–alginate scaffold, HepG2 cells display several differences as compared to 2D cultures or HCC cells grown on Matrigel, such as elevated angiogenic factors IL-8, bFGF, and VEGF, increased GPC-3 expression, low proliferation rate, and pronounced resistance to chemotherapy [40].

##### Bioprinted and 3D-Printed HCC Models

Bioprinted cell models are innovative platforms that enable the deposition of bioinks containing multiple types of living cells, signaling molecules, decellularized extracellular matrix constituents, nutrients, growth factors, and cell-laden biomaterials using a computer-aided design (CAD) in order to engineer 3D constructs with tissue-like architecture [98,104]. According to their origin, bioprinting materials vary from natural polymers such as alginate, gelatin, collagen, fibrin, and hyaluronic acid-based to synthetic materials [104].

Bioprinting technology is able to create constructs that effectively replicate the extracellular matrix, which enhances the attachment and proliferation of different cell types, including normal tissue-specific, connective tissues, and cancer cells [104]. When cultured in 3D bioprinted systems, tumor cells display an elevated proliferation rate and a better response to chemotherapy drugs as compared to conventional 2D models [105].

Xinwei Zhou’s group developed a 3D hepatic tumor platform consisting of HepG2 cells and a sodium alginate/gelatin/fibrinogen hydrogel as a model for effective drug screening. Their results revealed high cell viability and significant differences in their behavior after the treatment with anticancer drugs (5-fluorouracil, mitomycin) in comparison to 2D culture conditions [106].

##### HCC-on-a-Chip

Organ-on-a-chip modeling, which is the result of combining cell biology with engineering and biomaterial technology, stands among the most emerging technologies and has attracted enormous interest and attention lately. In brief, this method involves the construction of an organ biomimetic platform on a multi-channel microfluidic chip, recreating the structural and functional features of human physiology in terms of vascular and epithelial organ interfaces, as well as interactions between multiple organs [50,107]. The chip provides a microenvironment similar to that of the recreated organ [107]. Organ-on-a-chip models have four main components: (i) microfluidics; (ii) living cells (in 2D or 3D systems); (iii) stimulation; and (iv) sensing [107]. In particular, tumor-on-a-chip models add a new dimension to the mimicry of the human cancer microenvironment, offering great promise for the cost-effective and high-throughput evaluation of antineoplastic drugs [108].

Thus far, due to the central role of the liver in the metabolism of xenobiotics and its proneness to drug-induced toxicity [109], liver-on-a-chip platforms recapitulating the complex hepatic microenvironment have emerged as sophisticated microdevices in drug development [50].

Despite the challenges encountered when reconstructing the complex hepatic tissue, progress has been made so far. To mention a few recent studies, Lu and collaborators designed biomimetic liver tumors-on-a-chip by integrating decellularized liver matrixes with gelatin methacryloyl for a closer reflection of the 3D tumor microenvironment. Their platform exhibited a dose-dependent drug response to acetaminophen and sorafenib and represents an improved model for a range of future pharmacological and toxicological studies of anticancer drugs [107,110]. Sharif et al. developed an HCC–bone metastasis-on-a-chip system which models key aspects in the process of liver cancer invasion. This platform was used for studying the inhibitory effect of nanoparticle-encapsulated thymoquinone on the migration of HCC cells into the bone [111].

### 3.2. In Vivo Experimental Models for HCC

Animal models are well-established research tools used to study human diseases, building an important bridge between in vitro evaluations and clinical trials [112]. In the area of cancer research, accurate in vivo models offer substantial insights into the tumor biology in complex systems (living organisms) [113], disease etiology and pathology [114], and genetic mutations leading to the occurrence and development of tumors [113], facilitating the discovery of neoplastic drugs [112] by testing novel therapy approaches that cannot be safely used in patients [114], and predicting their response to therapy [115].

The development of accurate experimental animal models of liver tumors that are semblable to human hepatocarcinogenesis remains a challenge due to the complex etiology and tumor heterogeneity of HCC [116,117]. An ideal model should simulate the human HCC in terms of history, pathology, and biochemistry, allowing the analysis of potential drugs in preclinical studies and contributing to the progress of targeted therapy [116]. Despite the fact that no existing animal model completely captures the human disease [114], several valuable animal models have been designed so far for HCC. By offering several advantages, such as anatomical, physiological, and genetic resemblance to human beings, small size, large number of offspring, and low cost as compared to larger animals, rodent models represent the most preferred animal models for the research of HCC. In particular, mice have proven to be beneficial tools for the comprehension of the biological processes that occur during tumor growth, for preclinical evaluation of anticancer therapies [118], and for uncovering the molecular fundamentals of hepatocarcinogenesis [13]. A key factor in the selection of an animal model for studying HCC is represented by the strain of mice, since several strains have proven to be susceptible to developing this type of cancer spontaneously or chemically induced, such as C3H, CBA, and DBA/2 mice, whereas others are described as rather resistant—C57BL/6, BALB/c, and A/J. A detailed description of mouse strains’ susceptibility to HCC development can be found in two excellent articles [119,120].

Other relevant animal models for HCC research include the rat, woodchuck, and zebrafish. Further, several in vivo mouse and non-mouse models have been discussed.

#### 3.2.1. Mouse HCC Models

##### Chemically Induced HCC Mouse Models

Chemically induced models (CIM) are able to reveal underlying mechanisms of carcinogenesis by emphasizing the genetic, environmental, and immunological factors leading to cancer occurrence in the human population. The main disadvantages of CIM include the long time necessary for cancer induction and the unknown genetic background of the developed tumor, diminishing the use of these models lately. Nonetheless, the prolonged time is rather convenient because it facilitates the development of the inflammatory and fibrotic environmental features of human HCC [121].

**Figure 5:**
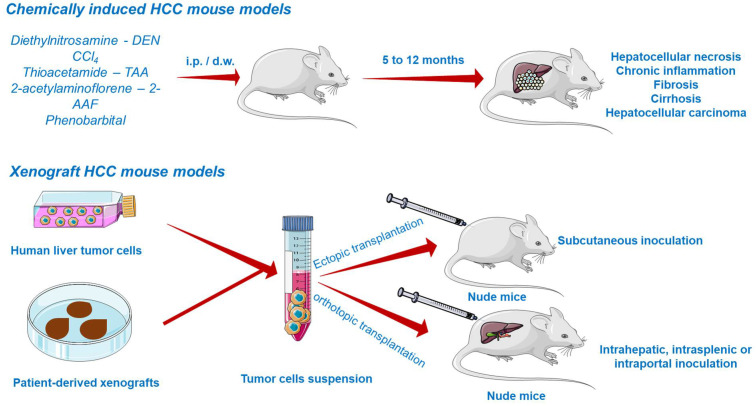
Chemically induced and xenograft mouse models of HCC. This image contains Servier Medical Art elements, which are licensed under a Creative Commons Attribution 3.0 Unported License; https://smart.servier.com, accessed on 20 June 2021.

Due to the inevitable daily life exposure to countless compounds exerting toxicity and to its fundamental role in xenobiotic detoxification, the liver is predisposed to severe damage [116]. Several chemicals able to induce liver injury are known so far and therefore applied in generating HCC models (Figure 5). They are primarily divided into (i) genotoxic compounds which alter the DNA structure and (ii) promoters able to enhance the formation of tumors [117,122]. Depending on the carcinogen type, chemically induced liver cancer is categorized as diethylnitrosamine (DEN)-, aflatoxin-, carbon tetrachloride-, dimethylnitrosamine-, and thioacetamide-induced hepatocarcinogenesis. Among these, DEN-induced liver carcinoma remains the most widely used model in preclinical research [123]. DEN targets the liver, where it is converted by cytochrome P450 enzymes present in centrilobular hepatocytes into alkylating metabolites that further induce DNA damage [7]. In addition, the contribution of DEN to hepatocarcinogenesis lies in its ability to cause oxidative stress [122]. The carcinogenic effect of DEN is dose-dependent, a single low dose being insufficient to form neoplasms due to the intervention of several DNA repair mechanisms, while the administration of a high dose induces HCC, but only after a period of latency. The time needed for the development of DEN-induced HCC is highly dependent on several factors such as the administered dose, sex, age, strain of mice [122], tumor microenvironment, and immune status [121]. HCC occurs faster in juvenile animals as a consequence of the high proliferation rate of hepatocytes [122]. An effective protocol involved the administration of a single DEN injection (1.25–25 mg/kg body weight) in two-week-old mice, resulting in the occurrence of liver cancer at around 8–12 months [124]. A conventional mouse model of DEN-induced liver carcinoma presumes the administration of a single injection of a low dose of DEN as an initiator. However, such a model does not develop the features of liver fibrosis, which is crucial to mimic tumor microenvironment of HCC in humans. The single injection of DEN can be accompanied by repeated dosing of CCl_4_ acting as a pro-fibrogenic agent in order to mimic the tumor microenvironment of HCC in humans. When injected chronically, DEN promotes inflammation and fibrosis, which accurately simulates the scenario of human HCC [121]. In a previous study conducted by Da Costa et al., a timeline of the evolution of DEN-induced HCC in mice has been sketched. According to the authors’ results, HCC developed in male ICR mice only 40 weeks after the first DEN intraperitoneal administration, showing specific histological features of malignancy. However, prior to HCC occurrence, several hepatic lesions were reported, such as hydropic degeneration, necrosis, apoptosis, hyperplastic foci, hepatocellular adenoma, and peliosis hepatis [125].

The DEN model is a reliable representation of HCC [122], due to the genetic resemblance, poor prognosis, high proliferation, low levels of β-catenin mutation, and apoptosis [124]. It can be applied in the study of the implication of immune responses and tumor microenvironment in the process of liver carcinogenesis [121]. Additionally, DEN-induced HCC in mice is a reliable model for drug and gene screening. Mohammed and colleagues used a DEN-induced HCC mouse model for evaluating the effect of nanoparticulate curcumin on HCC prophylaxis. They observed a significant elevation in hepatic enzymes, vascular endothelial growth factor (VEGF), tumor necrosis factor-α (TNF-α), α-fetoprotein, and nuclear factor-Kb (NF-Kb), as well as a reduced albumin concentration and tissular antioxidant activity following the treatment with DEN [126]. Luo et al. established a two-stage HCC model in BALB/c mice initiated with DEN and promoted with CCl_4_ and ethanol for simulating the molecular pathogenesis of human liver cancer by assessing the expression of proto-oncogenes and tumor suppressor gene p53 [127]. According to a recent study conducted by Connor et al., a single dose of DEN administered intraperitoneally to C3H/HeOuJ juvenile male mice (14–16 days old) determined several genetic aberrations, such as active mutations in *Hras*, *Braf*, *Egfr*, which act as oncogenic drivers, mutations that are seldom noticed in human HCC. In addition, there were observed *Apc*-truncated mutations that were correlated to an aberrant nuclear expression of β-catenin and an impairment of Wnt-β-catenin pathway, a specific feature of human HCC [7]. These data represent a reliable background for the selection of the most appropriate animal model when studying liver carcinogenesis.

##### Xenograft HCC Models 

The most common approach to establish HCC tumor xenograft models (Figure 5) is by the transplantation of biopsy material or cancer cell lines in the subcutaneous compartment or in the liver of immunodeficient mice [122,128]. Patient-derived tumors remain the first choice for generating realistic xenograft models because they retain the histological, molecular, and genetic heterogeneity of human cancers, while tumor cell lines hold a multitude of initial mutations and acquire several additional aberrations during in vitro culturing, distancing them from the features of cancers observed in patients, and poorly predicting the clinical outcome [129].

According to the implant site, xenografts can be divided into ectopic and orthotopic models. Ectopic xenografts, where the transplanted location differs from the origin of the cultured cells, represent the standard model used in oncology studies, allowing the easy monitoring of tumor growth, as well as the evaluation of cancer treatment efficacy [130]. In stark contrast to ectopic grafts, the orthotopic cancer models are advanced tools based on the implantation of human cancer cells in the same location as the origin tumor [130]. The orthotopic xenografts are superior due to their ability to replicate the tumor microenvironment, showing faster early-stage tumor growth, angiogenesis, and hyperpermeability of blood vessels [123,130]. Their major defect is the difficulty in monitoring the tumor growth and progression and measuring the tumor volume without animal sacrifice. However, to address this inconvenience, advanced imaging methods such as magnetic resonance imaging (MRI) or positron emission tomography (PET) are frequently applied to assess the evolution of tumors in orthotopic systems [123]. Xenograft platforms allow the preclinical investigation of pharmacokinetics, therapeutic efficacy, and toxicity of a compound [122,123].

Implantation HCC models that resemble the pre-existing liver conditions have been also described. Qi et al. successfully designed a novel HCC in vivo model that reflects the human liver cancer initiation and progression processes in the context of an existent hepatic injury (fibrosis, inflammation) through the inoculation of histologically normal oncogenic hepatocytes in C57BL/6J mice pre-treated with CCl_4_ as a fibrosis inducer [131].

Of particular interest are the implantable syngeneic mouse cancer models, which occupy a major position in tumor immunology and immunotherapy studies [132]. As compared to conventional xenografts, which require animal immunodeficiency, these models carry out the engraftment by subcutaneous or intravenous injection of mouse HCC cell lines or murine tumoral tissue in immunocompetent mice of the same genetic strain, while preserving the host immune system [132,133]. Reiberger and colleagues report the development of a syngeneic orthotopic HCC mouse model with CCl_4_-induced liver cirrhosis that mimics the features of human HCC [134].

##### Genetically Engineered HCC Mouse Models

Genetically engineered mouse models have made a substantial contribution to the field of oncology [129], playing a pivotal part in elucidating the mechanisms of tumor genesis, progression, therapeutic response, and innate drug resistance [135,136]. This method relies on the development in a natural non-immunodeficient environment of de novo tumors which closely reproduce the histological, pathological, and molecular heterogeneity of their human counterparts [129]. The most common methods for generating genetically tailored mouse cancer models are the activation/overexpression of oncogenes using tissue-specific promoters (e.g., KRAS), or the inactivation/removal of tumor suppressor genes via recombinase enzymes (e.g., CRISPR-Cas9 technology) [121,132].

Genetically modified models have become powerful research tools that provide insights into the involvement of specific proteins and signaling pathways in HCC development. Therefore, the primary advantage offered by these platforms is the knowledge of the initiating mutation, which plays a crucial role in the evaluation of molecularly targeted anti-HCC therapy options. Moreover, in these conditions, HCC arises spontaneously in a specific liver microenvironment, with an intact immune system. However, a disadvantage of these models is the absence of fibrosis and cirrhosis and reduced mutation burden, which is in contradiction to the human HCC, characterized by a landscape of altered genes and pathways, as well as a pre-existing liver disease [121].

In order to better understand the implication of cancer-related genes in HCC development [137], there is a large number of representative genetically engineered mice for liver cancer, from models overexpressing oncogenes (e.g., Myc, β-catenin) [122] and growth factors (e.g., EGF, TGF-α, FGF19) [122] to rodents presenting inactivated/removed tumor suppressor genes (e.g., p53) [138]. However, experimental research has shown that alterations within a single gene are generally insufficient for inducing hepatocarcinogenesis, which is, in essence, a multistep process requiring multiple mutations [137]. For instance, transgenic mice overexpressing c-Myc or β-catenin alone inefficiently develop HCC within a long latent period, while additional oncogenes or growth factors generate early liver cancer. Similarly, HCC induced via p53 inactivation is accelerated when supplementary oncogenes are expressed [137].

Sook In Chung’s research group developed a transgenic HCC in vivo model by applying the hydrodynamic transfection method and the Sleeping Beauty transposon system, which induced concomitant c-Myc expression and p53 suppression in 5-to-6-week-old C57BL/6 male mice. Additionally, they combined this technique with CCl_4_ treatment, creating a liver fibrosis background which significantly accelerated the hepatocarcinogenesis induced by c-Myc up- and p53 downregulation [139].

##### Humanized HCC Mouse Models

Humanized models (HMs) are generated by engrafting human tumor cells or patient-derived xenografts into an immunodeficient rodent host harboring constituents of the human immune system. These novel platforms emerged as a step forward in the preclinical cancer research by reproducing the realistic interactions occurring between the tumor and the immune system [140,141], which make a significant contribution to cancer progression [132]. Regarding the importance of inducing immunodeficiency in mice prior to inoculation, its main role is to facilitate the engraftment and to overcome the rejection of human cancer cells mediated by the murine immune system [140].

The in vivo reconstitution of the human immune system can be acquired by (i) the intravenous transplantation of human peripheral blood mononuclear cells (PBMCs), which generates the so-called PBL (peripheral blood lymphocyte)-HMs, (ii) the inoculation of CD34+ hematopoietic stem cells (HSCs) leading to the HSC/SRC/SCID-HMs [142], which provides a more complete immune restoration, or (iii) the sub-renal administration of human fetal thymus or fetal liver tissue, followed by the injection of autologous CD34+ HSCs, resulting in the BLT (bone marrow–liver–thymus)-HMs [140,143].

Humanized mouse models are mainly applied in the investigation of oncologic immunotherapy [144]. Unfortunately, despite being one of the most attractive preclinical models for immunotherapy screening, humanized rodents are currently not well established in the area of HCC study. However, advances have been made in this regard. For instance, Zhao and colleagues designed a new patient-derived xenograft humanized mouse model to study the interactions between HCC and the human immune system, and to evaluate and predict the efficacy of immunotherapy and combination therapies [145]. Similarly, Bi et al. established a novel patient-derived xenograft model of human HCC in immunocompetent mouse [146].

#### 3.2.2. Non-Mouse HCC Models

##### Rat HCC Model 

The laboratory rat (*Rattus norvegicus*) is a rodent model that has been faithfully used in several biomedical domains, including oncology [147]. In particular, experimental rat hepatocarcinogenesis served for a long time as a relevant model in delineating the pathogenesis of liver neoplasia, evaluating the human cancer development risk following exposure to carcinogens [148], facilitating HCC prevention strategies [149], identifying the metabolic pathways leading to liver tumorigenesis [150], and evaluating the tumor response to chemotherapy [151].

##### Woodchuck HCC Model

The eastern woodchuck (*Marmota monax*) is acknowledged as a clinically relevant in vivo experimental model in the investigation of HBV-associated diseases such as HCC [152]. This model spontaneously develops chronic hepatitis advancing to HCC during adulthood after neonatal exposure to woodchuck hepatitis virus (WHV) [153,154]. Several similarities have been established between WHV and human HBV with respect to the viral life cycles, mechanisms of infection and replication, nucleotide sequence, genome organization, and virion morphology [154,155]. Following the inoculation, liver cancer development occurs within an approximate period of 24–32 months [153].

Not only does woodchuck HCC resemble the human HCC in regard to imaging appearance and biological features [156], but it also is recognized as a unique system that effectively models several events of HBV-associated liver cancer, such as viral infection, leading to the subsequent HCC development and generation of immune reactions against the virus and the tumor [154]. Thus far, woodchucks with established HCC tumors have been used in various applications. As compared to mice or rats, which are limited by their small size, this particular model is ideal in the evaluation of HCC intra-arterial therapies [153]. Additionally, HCC-bearing woodchucks have been effectively used in the evaluation of novel therapeutic strategies, improvement of current imaging and ablation techniques, and prevention of HCC initiation and progression [155].

##### Zebrafish HCC Model

Over the last few decades, zebrafish (*Danio rerio*)—a vertebrate model organism [157,158]—have arisen as a faithful platform in the field of drug discovery and toxicity evaluation due to their organ similarities to mammals. Zebrafish models possess a plethora of unique advantages, including the generation of a large number of progeny, fast development, small size, confidence in statistical analysis, feasible drug administration, low-cost testing as compared to other animals, high molecular and genetic homology to human beings, generation of cancer models that share similarities to their human counterparts, as regards the molecular and pathological aspects, and optical transparency during the first days of life, which permits the real-time non-invasive observation of tumor metastasis, efficacy, and toxicity of the antitumoral drugs [157,159,160,161]. In addition, larval zebrafish are considered a useful replacement for some animal toxicity studies, allowing an early identification of toxic molecules, the evaluation of safer compounds in mammals, and the reduction of the number of animals used in the study [161].

Due to the high resemblance in genes, molecular pathways, and response to drug treatment, zebrafish became popular among the models used to study human cancer biology, offering a complex view on carcinogenesis and cancer metastasis, the tumor microenvironment, and angiogenesis, as well as drug screening, toxicity, and resistance [157,162]. Cancer can be induced in zebrafish by three methods: (i) exposure to chemical carcinogens (e.g., ethylnitrosourea and N-methyl-nitrosoguanidine able to initiate the development of various malignancies), (ii) generation of mutations in oncogenes or tumor suppressor genes (e.g., TP53, which is the most commonly altered tumor suppressor gene in human cancers), and (iii) xenotransplantation, resulting in a wide array of tumors in different organs [158,162].

In particular, zebrafish represent a useful system for assessing the mechanisms of hepatic diseases, including hepatocellular carcinoma, due to the wide similarities shared between their liver and the human liver at both physiological and pathological levels [13,163]. Several transgenic zebrafish models have been developed to mimic HCC, including β-Catenin-, Xmrk (EGFR)-, KRAS-, and Myc-driven models [164]. Nguyen et al. generated a transgenic zebrafish model expressing EGFP-kras^V12^ by administrating mifepristone for the high-throughput screening of new cancer targets or inhibitors of Ras-mediated hepatocarcinogenesis [165].

### 3.3. Computational Modeling of HCC

#### 3.3.1. In Silico Models

In silico experimentation refers to the coupling of current computer-based technologies with conventional biology, providing a guided and targeted approach in the study of cancer. Unlike traditional laboratory-based research, which involves expensive and time-consuming experimentation on biological materials (e.g., cell cultures) or animals to investigate hypotheses or even make predictions about the treatment effectiveness, by means of specifically designed computer programs, in silico modeling offers the possibility to simulate the real experimental environments by conducting computational experiments [147]. In other words, in silico experimentation plays a complementary role to conventional in vitro and in vivo modeling, their interplay being vital for the progress of research [166,167]. In fact, the in silico method is an extrapolation of in vitro and in vivo studies [168], being based on the use of data obtained from past preclinical experiments [169]. The main benefit provided by in silico models is the meeting of the 3R (i.e., replacement, reduction, refinement) principles, by which not only the number of animals used in in vivo studies is reduced but also the costs of laboratory experimentation [166].

A particular domain for in silico modeling is represented by drug design and development, which is essential in the case of HCC, a domain that lacks curable chemotherapy options. Generally, this computational approach is applied in virtual ligand screening and affinity profiling, improving the discovery of active compounds presenting an affinity for a specific molecular target by prioritizing molecules and targets for further in vitro and in vivo validation [167]. For instance, by performing an in silico study via the Connectivity Map (CMap) database, Liu et al. predicted the potential of sorafenib to inhibit the activity of histone deacetylase (HDAC), which has been further confirmed in vitro in both sorafenib-sensitive and -resistant hepatocellular carcinoma cells [170]. Notably, the repurposing of the currently FDA-approved compounds stands as a strategic approach in oncologic drug discovery, in silico modeling playing a significant part in simplifying this process. As an example, Shi and colleagues utilized the idock software to specifically identify nine compounds exerting a potential anti-HCC effect, among which the anti-psychotic medicine fluspirilene demonstrated the strongest antiproliferative activity against HepG2 and HuH-7 cells in vitro and a HCC tumor growth-inhibiting property in Balb/c mice bearing HuH-7 xenografts [171].

Moreover, this technique has been reported to be useful in the identification of crucial biomarkers and specific pathways in HCC. For instance, Sun et al. applied in silico methods to identify a significant gene—DEP domain-containing protein 1B (DEPDC1B)—which might be a novel biomarker for the diagnosis and prognosis of HCC [172]. In another study, computational methods able to simulate drug–receptor interactions were applied in order to explore the best therapeutic candidates in the treatment of HCC. Taking into consideration the Bcl-XL and FGF proteins as molecular targets for apoptosis and angiogenesis, respectively, the author identified two eligible candidates for FGF and one candidate for Bcl-XL [173].

#### 3.3.2. Prediction Models of HCC Using Artificial Intelligence and Machine Learning Methods

The concept of artificial intelligence (AI)—dating back to the late 1950s [174]—represents a field of computer science [175] that aims to design machines able to accomplish complex tasks typically associated with human intellect features such as reasoning and thinking powers [174,176]. By definition, artificial intelligence refers to the use of computerized mathematical algorithms to recreate human cognitive behavior and intelligence, which can be utilized for solving difficult challenges, the AI-based techniques being lately employed with great interest in healthcare research areas, including cellular biology, drug discovery and formulation, clinical diagnosis, and disease treatment [177,178,179]. In particular, AI algorithms hold tremendous potential within the field of oncology, paving the pathway that leads to the identification of genetic mutations at an early stage, and they allow the diagnosis and the efficient evaluation of cancer prognosis, progression, treatment, and susceptibility to recurrence [174,177]. Furthermore, AI methods represent a rapid and low-cost alternative to the current oncology drug discovery, which is, in essence, expensive and time-consuming as a consequence of the multitude of candidates failing during the late development stages [180].

Machine learning (ML) is a branch or subset of artificial intelligence [177,181] that has made a prominent contribution to cancer research and practice by providing an in-depth tumor understanding and, accordingly, the premise of personalized and efficient oncologic care [176]. ML platforms consist of a set of data points that are trained via mathematical and statistical methods to enable the prediction of novel data without explicit programming [182]. In other words, the rules and logic are not predetermined, but learned by the program via continuous exposure to data [183]. ML algorithms are typically classified into supervised, unsupervised, and reinforcement learning [182]. Supervised learning comprises self-improving models with data that are associated with a known result, and it is divided in two categories—classification and regression—which permit the distinction between cancer types and the assessment of the tumor response to a particular therapy approach, respectively [176,183]. In contrast, unsupervised learning is applied in the situation of an unknown outcome or when the discovery of new data patterns is desired [176], whilst reinforcement learning shares common features with the two previously described learning methods, being less employed in pathology studies [182]. ML algorithms provide several advantages over traditional methods, such as the ability to process big and non-linear data, high predictive performance by learning from existing data, and high-speed data processing. However, shortcomings such as challenging algorithm selection, lack of standards for ML model design, and perfect generalization capability should be taken into account [184].

The AI approach stands as an ideal strategy for HCC modeling, performing a combined evaluation of clinical, histological, and radiological data that predicts numerous outcomes such as cancer diagnosis, pathological features, treatment response, and survival rate, which, in real life, encounter difficulties due to the heterogeneous nature of the disease [179,185,186]. In a comprehensive review by Lai and colleagues, it has been brought to the spotlight that the majority of AI studies (60%) focus on HCC diagnosis [179]. Sato et al. designed a novel graphical user interface machine learning framework as a predictive tool for accurate HCC diagnosis by using real-life data obtained from HCC patients during clinical practice [187].

AI plays a significant role in HCC therapy by offering predictions regarding the tumor response to treatment, thus allowing the accurate selection of the most suitable option [186]. By associating magnetic resonance imaging with clinical data, Abajian et al. developed an ML-based framework for the pre-procedural prediction of HCC patients’ therapeutic outcome after trans-arterial chemoembolization, showing 78% accuracy [188]. Similarly, by using quantitative CT imaging, Morshid and collaborators designed a fully automated ML model which recreates the HCC response to transcatheter arterial chemoembolization, with a prediction accuracy rate of 74.2% [189].

Due to the inadequate anticipation of the survival and recurrence rates in HCC patients following treatment, AI models have been employed in this area of liver cancer research as well [179,190]. Huang et al. developed and validated an ML prognostic model to select high-risk HCC patients after surgical resection at four time intervals ranging from 0 to 5 years following the procedure. Additionally, the authors constructed a risk heat map which offers visual insight into the HCC recurrence risk in different years [190]. Saito et al. designed a model for predicting the early recurrence of HCC based on images of hematoxylin–eosin (HE)-stained specimens and ML using a support vector machine, which offered a 89.9% accuracy rate [191].

## 4. Future Perspectives

A novel concept that could provide insightful data concerning tumor growth, metastatic process, and response/resistance to treatment in HCC is represented by liver cancer stem cells (CSC). These cells are a subset of cancer cells that retain the stem cells’ characteristics, such as self-renewal capacity and irregular division, generating heterogenous cellular populations capable of escaping cell apoptosis and developing resistance to treatment [192]. To date, there have been discovered two categories of liver CSC: (i) Ep-CAM positive, which are highly tumorigenic and present epithelial-like features, and (ii) CD90-positive, with high metastatic potential and mesenchymal phenotype [192]. The available evidence regarding liver CSC explains the heterogeneous character of HCC concerning tumor pathogenesis and treatment response, but further efforts are needed to comprehend the utility of these cells in establishing clinical diagnostic and novel therapeutic approaches.

## 5. Conclusions

Despite the breakthroughs recorded in the field of HCC in terms of understanding the pathogenesis, molecular mechanisms, genetics, and therapeutical approaches, this pathology still represents a global concern and a burden for health systems worldwide.

In recent years, preclinical in vitro and in vivo models proved to be valuable and reliable tools for gathering insights about HCC, but contrary to the achieved progress, several facets of human HCC remain undiscovered. However, a single model able to recreate the integral landscape of genetic and cellular characteristics of HCC represents, at present, an infeasible task. Nevertheless, by carefully combining the data provided by the array of in vitro, in vivo, and computational techniques, we could obtain a tailored experimental model that bridges the gap between the basic research and clinical application.

## Figures and Tables

**Table 1:** Presentation of the cell lines currently described in the literature as established in vitro experimental models for HCC.

Cell Line	Origin	Disease	Sensitivity to HCC Chemotherapy (Sorafenib)	Frequency (No. of PubMed Studies)	Applications
HepG2	*Homo sapiens*, 15-year-old male [56]	HCC	IC_50_ = 6 µM [57]; IC_50_ = 7.42 µM [58]	32,929	3D modeling; cancer research; toxicology studies; high-throughput screening [56]
Hep3B	*Homo sapiens*, 8-year-old juvenile male [56]	HCC	IC_50_ = 3.31 µM [58]	2908	3D cell culture; high-throughput screening; cancer research; infectious and sexually transmitted disease research; toxicology evaluations [56]
HuH-7	*Homo sapiens*, 57-year-old male [48]	Well-differentiated HCC	IC_50_ = 5.97 µM [58]	2545	3D modeling [11]; drug testing [59,60] and repurposing [61]; drug metabolism studies [62]
C3A	*Homo sapiens*, 15-year-old male [48]	Differentiated HCC	-	2070	3D cultures and cancer research [56]
SKHep1	*Homo sapiens*, 52-year-old male [48]	Adenocarcinoma	IC_50_ = 5.3 ± 0.5 µM [63]	976	3D modeling; cancer research; toxicology studies; high-throughput screening; cardiovascular disease research [56]
HepaRG	*Homo sapiens*, female patient [52]	Tumor from the liver of a female diagnosed with chronic hepatitis C and macronodular cirrhosis [54]	-	835	Bioartificial liver application [64]; in vitro drug metabolism and toxicology evaluations [65]; 3D model design [66]
Hepa1-6	*Mus musculus*, C57L mouse strain [56]	Hepatoma	Effective concentrations = 10–50 µM [67]	386	3D cultures and cancer research [56]
LMH	*Gallus gallus*, Leghorn strain chicken [56]	chemically induced HCC	-	321	3D cultures and cancer research [56]
SNU-475	*Homo sapiens*, 43-year-old male [56]	grade II–IV/V HCC	Effective concentrations = 20–50 µM [68]	20	3D modeling; infectious disease research; sexually transmitted disease research; cancer research [56]
SNU-387	*Homo sapiens*, 41-year-old female [56]	grade IV/V pleomorphic HCC	Effective concentrations = 10–50 µM [68]	17	3D modeling; infectious disease research; sexually transmitted disease research; cancer research [56]

**Table 2:** Examples of in vitro 3D models of HCC.

In Vitro 3D Model	Cell Line(s)	Observations	Reference
Co-culture on polycaprolactone electrospun scaffolds	HepG2 and patient-derived human healthy hepatocytes (HHH)	antiproliferative and antioxidant activities of the scaffold in the case of HepG2 cells and their co-culture with HHH	[73]
Co-culture on double-layered fibrous scaffolds incorporated with hydrogel micropatterns	HepG2 spheroids and fibroblasts	↑ albumin secretion	[74]
Co-culture	HuH-7 and LX2	induced drug (sorafenib) resistance in HCC cells by HGF/c-Met/Akt and Jak2/Stat3 signaling pathways	[72]
Co-culture	HepaRG and LX2	increased expression of proinflammatory cytokines; ↑ VEGFA and matrix metalloproteinase-9 expression in hepatic stellate cells; permissive proangiogenic microenvironment	[75]
Co-culture	HuH-7 spheroids and human umbilical vein endothelial cells (HUVEC)	↑ proliferation and gene expression of HCC-related genes; activation of the epithelial–mesenchymal transition (EMT) and angiogenic pathways; ↑ angiogenesis and vessel maturation	[76]
Spheroids	HuH-7	activation of apoptotic and proliferative HIF-1α and ERK signals	[76]
Spheroids	HepG2	experimental model for genotoxicity assessment	[77]
Organoid-like spheroids in porous alginate scaffolds	HuH-1, HuH-7, HepG2, Hep3B, SK-Hep-1	↑ sensitivity to TGF/β-induced EMT; ↑ in vivo tumorigenic and metastatic potential; ↑ resistance to chemotherapeutic drugs as compared to 2D cultures	[78]
Tumor Organoid System	HCCLM3, Hep3B, HUVEC, and human primary fibroblasts	similar features to human HCC observed in vivo; ↑ neo-angiogenesis-related markers (VEGFR2, VEGF, HIF-a), tumor-related inflammatory factors (CXCR4, CXCL12, TNF-a epithelial–mesenchymal transition markers (TGFb, Vimentin, MMP9)	[79]
Bioprinted Model	HepG2, NIH 3T3	↑ adhesion, viability, proliferation, function	[80]
Bioprinted Model	HepG2	↑ expression of tumor-related genes, differences in drug resistance genes as compared to 2D model	[81]
Cirrhotic decellularized ECM Scaffold Based Bioprinted Model	HepG2	↓ cell growth; ↑ invasion markers (matrix metalloproteinases MMP2 and MMP9, Twist-related protein 1)	[82]

↑ increase, ↓ decrease.

**Table 3:** A summary characterization of 3D in vitro models.

Type of 3D In Vitro Model	Specific Features	Biomedical Applications	Reference(s)
Co-cultures	direct—different cells are cultured together indirect—the cells are separated by a physical barrier cells of different phenotypes do not grow together even when they are in direct contact	Cell–cell communication Cell–microenvironment interactions Cancer invasion	[89]
Spheroids	non-scaffold-based 3D model round-shaped micro-sized cellular aggregates can be generated either from 2D cell cultures (primary or immortalized cells) or tissue fragments multilayer structure: (i) external layer—proliferative cells, (ii) middle layer—senescent cells, and (iii) core—necrotic cells two types of tumor spheroids: homodymic, containing ex-clusively cancer cells, and heterodymic, containing tumor cells cul-tivated with other cell types ECM consists of proteins produced by the cells during sphe-roid formation	Drug discovery Disease modelling Toxicity screening High-throughput screening	[90,91,92]
Organoids	also known as “organ buds” complex scaffold-free 3D models stem cell organoids (derived from embryonic stem cells/induced pluripotent cells/primary stem cells) tissue organoids (stromal cell-free culture) ‘organ-in-a-dish’ in vivo-like complexity and architecture	Carcinogenesis studies Anticancer drug screen-ing and discovery Development of person-alized anticancer therapies	[12,37,90,92,93,94,95]
Scaffold-based models	3D constructs providing a physical support (matrix) on which cells can proliferate, divide, and migrate specifically designed to recapitulate the in vivo ECM composed of natural (i.e., Matrigel, chitosan, hyaluronic acid, alginate) or synthetic (i.e., polyethylene glycol, polyvinyl alcohol) scaffolds	Drug screening and dis-covery	[92,96,97]
Bioprinted and 3D printed models	layer-by-layer deposed bioinks (i.e., cell pellets; decellularized ECM constituents) with 3D architecture scaffold-free design—bioprinting on sacrificial materials (agarose; alginate), which are eventually discarded, or scaffold-based design—bioprinting of hydrogel-encapsulated bioinks	Tissue engineering Cancer pathology re-search Anticancer drug discov-ery	[97,98]
Organ-on-a-chip	miniature microfluidic devices made of optically clear mate-rials (i.e., plastic, glass, polymers) containing microchannels popu-lated by living cells extended viability of the cultured cells (weeks, months) faithful simulation of the in vivo organ structure and func-tions mimicking both the physiological and pathological features of the organ	Modeling of specific tumoral processes: growth, neovascularization and angiogenesis, progression from early to late stages, invasion, and metastasis	[92,99]

## References

[1] Forner A., Reig M., Bruix J. (2018). Hepatocellular carcinoma. Lancet.

[2] Rebouissou S., Nault J.C. (2020). Advances in molecular classification and precision oncology in hepatocellular carcinoma. J. Hepatol..

[3] Molina-Sánchez P., Lujambio A., Hoshida Y. (2019). Experimental Models for Preclinical Research in Hepatocellular Carcinoma. Hepatocellular Carcinoma: Translational Precision Medicine Approaches.

[4] Hirschfield H., Bian C.B., Higashi T., Nakagawa S., Zeleke T.Z., Nair V.D., Fuchs B.C., Hoshida Y. (2018). In vitro modeling of hepatocellular carcinoma molecular subtypes for anti-cancer drug assessment. Exp. Mol. Med..

[5] Villanueva A. (2019). Hepato-cellular carcinoma. N. Engl. J. Med..

[6] Newell P., Villanueva A., Friedman S.L., Koike K., Llovet J.M. (2008). Experimental models of hepatocellular carcinoma. J. Hepatol..

[7] Connor F., Rayner T.F., Aitken S.J., Feig C., Lukk M., Santoyo-Lopez J., Odom D.T. (2018). Mutational landscape of a chemically-induced mouse model of liver cancer. J. Hepatol..

[8] Ghavimi S., Apfel T., Azimi H., Persaud A., Pyrsopoulos N.T. (2020). Management and treatment of hepatocellular carcinoma with immunotherapy: A review of current and future options. J. Clin. Transl. Hepatol..

[9] Neureiter D., Stintzing S., Kiesslich T., Ocker M. (2019). Hepatocellular carcinoma: Therapeutic advances in signaling, epigenetic and immune targets. World J. Gastroenterol..

[10] Piñero F., Silva M., Iavarone M. (2020). Sequencing of systemic treatment for hepatocellular carcinoma: Second line competitors. World J. Gastroenterol..

[11] Khawar I.A., Park J.K., Jung E.S., Lee M.A., Chang S., Kuh H.J. (2018). Three Dimensional Mixed-Cell Spheroids Mimic Stroma-Mediated Chemoresistance and Invasive Migration in hepatocellular carcinoma. Neoplasia.

[12] Fan H., Demirci U., Chen P. (2019). Emerging organoid models: Leaping forward in cancer research. J. Hematol. Oncol..

[13] Yim S.Y., Shim J.J., Sohn B.H., Lee J.S., Irwin M.A., Harvey J.A., James L.B., David E.C., David A.S., Snorri S.T., Allan W. (2020). Experimental Models of Liver Cancer: Genomic Assessment of Experimental Models. The Liver: Biology and Pathobiology.

[14] Grandhi M.S., Kim A.K., Ronnekleiv-Kelly S.M., Kamel I.R., Ghasebeh M.A., Pawlik T.M. (2016). Hepatocellular carcinoma: From diagnosis to treatment. Surg. Oncol..

[15] Ghouri Y.A., Mian I., Rowe J.H. (2017). Review of hepatocellular carcinoma: Epidemiology, etiology, and carcinogenesis. J. Carcinog..

[16] Geh D., Anstee Q.M., Reeves H.L. (2021). NAFLD-Associated HCC: Progress and Opportunities. J. Hepatocell. Carcinoma.

[17] Farci P., Niro G.A., Zamboni F., Diaz G. (2021). Hepatitis D virus and hepatocellular carcinoma. Viruses.

[18] Clark T., Maximin S., Meier J., Pokharel S., Bhargava P. (2015). Hepatocellular Carcinoma: Review of Epidemiology, Screening, Imaging Diagnosis, Response Assessment, and Treatment. Curr. Probl. Diagn. Radiol..

[19] Malik A., Thanekar U., Amarachintha S., Mourya R., Nalluri S., Bondoc A., Shivakumar P. (2021). “Complimenting the Complement”: Mechanistic Insights and Opportunities for Therapeutics in Hepatocellular Carcinoma. Front. Oncol..

[20] Bozward A.G., Warricker F., Oo Y.H., Khakoo S.I. (2021). Natural Killer Cells and Regulatory T Cells Cross Talk in Hepatocellular Carcinoma: Exploring Therapeutic Options for the Next Decade. Front. Immunol..

[21] Vij M., Calderaro J. (2021). Pathologic and molecular features of hepatocellular carcinoma: An update. World J. Hepatol..

[22] Schulze K., Nault J.C., Villanueva A. (2016). Genetic profiling of hepatocellular carcinoma using next-generation sequencing. J. Hepatol..

[23] Niu Z.S., Niu X.J., Wang W.H. (2016). Genetic alterations in hepatocellular carcinoma: An update. World J. Gastroenterol..

[24] Nault J.C., Zucman-Rossi J. (2016). TERT promoter mutations in primary liver tumors. Clin. Res. Hepatol. Gastroenterol..

[25] Inokawa Y., Inaoka K., Sonohara F., Hayashi M., Kanda M., Nomoto S. (2016). Molecular alterations in the carcinogenesis and progression of hepatocellular carcinoma: Tumor factors and background liver factors (Review). Oncol. Lett..

[26] Khalaf A.M., Fuentes D., Morshid A.I., Burke M.R., Kaseb A.O., Hassan M., Hazle J.D., Elsayes K.M. (2018). Role of Wnt/β-catenin signaling in hepatocellular carcinoma, pathogenesis, and clinical significance. J. Hepatocell. Carcinoma.

[27] Galicia-Moreno M., Silva-Gomez J.A., Lucano-Landeros S., Santos A., Monroy-Ramirez H.C., Armendariz-Borunda J. (2021). Liver Cancer: Therapeutic Challenges and the Importance of Experimental Models. Can. J. Gastroenterol. Hepatol..

[28] Wang H., Yang J., Zhang K., Liu J., Li Y., Su W., Song N. (2021). Advances of Fibroblast Growth Factor/Receptor Signaling Pathway in Hepatocellular Carcinoma and its Pharmacotherapeutic Targets. Front. Pharmacol..

[29] Tenen D.G., Chai L., Tan J.L. (2021). Metabolic alterations and vulnerabilities in hepatocellular carcinoma. Gastroenterol. Rep..

[30] van Tienderen G.S., Koerkamp B.G., Ijzermans J.N.M., van der Laan L.J.W., Verstegen M.M.A. (2019). Recreating tumour complexity in a dish: Organoid models to study liver cancer cells and their extracellular environment. Cancers.

[31] Sevic I., Spinelli F.M., Cantero M.J., Reszegi A., Kovalszky I., García M.G., Alaniz L., Tirnitz-Parker J. (2019). The Role of the Tumor Microenvironment in the Development and Progression of Hepatocellular Carcinoma. Hepatocellular Carcinoma.

[32] Chedid M.F., Kruel C.R.P., Pinto M.A., Grezzana-Filho T.J.M., Leipnitz I., Kruel C.D.P., Scaffaro L.A., Chedid A.D. (2017). Hepatocellular Carcinoma: Diagnosis and Operative Management. ABCD Arq. Bras. Cir. Dig..

[33] Zhao Y., Zhang Y.N., Wang K.T., Chen L. (2020). Lenvatinib for hepatocellular carcinoma: From preclinical mechanisms to anti-cancer therapy. Biochim. Biophys. Acta Rev. Cancer.

[34] Costa E., Ferreira-Gonçalves T., Chasqueira G., Cabrita A.S., Figueiredo I.V., Reis C.P. (2020). Experimental models as refined translational tools for breast cancer research. Sci. Pharm..

[35] Katt M.E., Placone A.L., Wong A.D., Xu Z.S., Searson P.C. (2016). In vitro tumor models: Advantages, disadvantages, variables, and selecting the right platform. Front. Bioeng. Biotechnol..

[36] Mirabelli P., Coppola L., Salvatore M. (2019). Cancer cell lines are useful model systems for medical research. Cancers.

[37] Saydé T., Hamoui O.E., Alies B., Gaudin K., Lespes G., Battu S. (2021). Biomaterials for three-dimensional cell culture: From applications in oncology to nanotechnology. Nanomaterials.

[38] Kimlin L.C., Casagrande G., Virador V.M. (2013). In vitro three-dimensional (3D) models in cancer research: An update. Mol. Carcinog..

[39] Duval K., Grover H., Han L.H., Mou Y., Pegoraro A.F., Fredberg J., Chen Z. (2017). Modeling physiological events in 2D vs. 3D cell culture. Physiology.

[40] Leung M. (2011). Chitosan-Alginate Scaffold Culture System for Hepatocellular Carcinoma Increases Malignancy and Drug Resistance. Bone.

[41] Chiew G.G.Y., Wei N., Sultania S., Lim S., Luo K.Q. (2017). Bioengineered three-dimensional co-culture of cancer cells and endothelial cells: A model system for dual analysis of tumor growth and angiogenesis. Biotechnol. Bioeng..

[42] Bartlett R., Everett W., Lim S., Natasha G., Loizidou M., Jell G., Tan A., Seifalian A.M. (2014). Personalized in vitro cancer modeling—Fantasy or reality?. Transl. Oncol..

[43] Koledova Z. (2017). 3D Cell Culture: An Introduction. Methods Mol. Biol..

[44] Wang C., Tang Z., Zhao Y., Yao R., Li L., Sun W. (2014). Three-dimensional in vitro cancer models: A short review. Biofabrication.

[45] Ravi M., Paramesh V., Kaviya S.R., Anuradha E., Paul Solomon F.D. (2015). 3D cell culture systems: Advantages and applications. J. Cell. Physiol..

[46] Collins S.D., Yuen G., Tu T., Budzinska M.A., Spring K., Bryant K., Shackel N.A., Tirnitz-Parker J. (2019). In Vitro Models of the Liver: Disease Modeling, Drug Discovery and Clinical Applications. Hepatocellular Carcinoma.

[47] Qiu Z., Zou K., Zhuang L., Qin J., Li H., Li C., Zhang Z., Chen X., Cen J., Meng Z. (2016). Hepatocellular carcinoma cell lines retain the genomic and transcriptomic landscapes of primary human cancers. Sci. Rep..

[48] Fukuyama K., Asagiri M., Sugimoto M., Tsushima H., Seo S., Taura K., Uemoto S., Iwaisako K. (2021). Gene expression profiles of liver cancer cell lines reveal two hepatocyte-like and fibroblast-like clusters. PLoS ONE.

[49] Qiu G.H., Xie X., Xu F., Shi X., Wang Y., Deng L. (2015). Distinctive pharmacological differences between liver cancer cell lines HepG2 and Hep3B. Cytotechnology.

[50] Deng J., Wei W., Chen Z., Lin B., Zhao W., Luo Y., Zhang X. (2019). Engineered liver-on-a-chip platform to mimic liver functions and its biomedical applications: A review. Micromachines.

[51] Vinken M., Rogiers V. (2015). Culture and Functional Characterization of Human Hepatoma HepG2 Cells María. Protoc. Vitr. Hepatocyte Res..

[52] Mavri-Damelin D., Damelin L.H., Eaton S., Rees M., Selden C., Hodgson H.J. (2008). Cells for bioartificial liver devices: The human hepatoma-derived cell line C3A produces urea but does not detoxify ammonia. Biotechnol. Bioeng..

[53] van Wenum M., Adam A.A.A., Hakvoort T.B.M., Hendriks E.J., Shevchenko V., van Gulik T.M., Chamuleau R.A.F.M., Hoekstra R. (2016). Selecting cells for bioartificial liver devices and the importance of a 3D culture environment: A functional comparison between the hepaRG and C3A cell lines. Int. J. Biol. Sci..

[54] Goyak K.M.O., Laurenzana E.M., Omiecinski C.J. (2010). Hepatocyte Differentiation. Methods Mol. Biol..

[55] Lacoste B., Raymond V.A., Cassim S., Lapierre P., Bilodeau M. (2017). Highly tumorigenic hepatocellular carcinoma cell line with cancer stem cell-like properties. PLoS ONE.

[56] ATCC:The Global Bioresource Center. https://www.atcc.org/.

[57] Wei J.C., Meng F.D., Qu K., Wang Z.X., Wu Q.F., Zhang L.Q., Pang Q., Liu C. (2015). Sorafenib inhibits proliferation and invasion of human hepatocellular carcinoma cells via up-regulation of p53 and suppressing FoxM1. Acta Pharmacol. Sin..

[58] Liu J., Liu Y., Meng L., Ji B., Yang D. (2017). Synergistic antitumor effect of sorafenib in combination with ATM inhibitor in hepatocellular carcinoma cells. Int. J. Med. Sci..

[59] Yang C., Qin S. (2018). Apatinib targets both tumor and endothelial cells in hepatocellular carcinoma. Cancer Med..

[60] Hoshi T., Watanabe Miyano S., Watanabe H., Sonobe R.M.K., Seki Y., Ohta E., Nomoto K., Matsui J., Funahashi Y. (2019). Lenvatinib induces death of human hepatocellular carcinoma cells harboring an activated FGF signaling pathway through inhibition of FGFR–MAPK cascades. Biochem. Biophys. Res. Commun..

[61] Shi T., Fujita K., Gong J., Nakahara M., Iwama H., Liu S., Yoneyama H., Morishita A., Nomura T., Tani J. (2020). Aspirin inhibits hepatocellular carcinoma cell proliferation in vitro and in vivo via inducing cell cycle arrest and apoptosis. Oncol. Rep..

[62] Lin J., Schyschka L., Mühl-Benninghaus R., Neumann J., Hao L., Nussler N., Dooley S., Liu L., Stöckle U., Nussler A.K. (2012). Comparative analysis of phase I and II enzyme activities in 5 hepatic cell lines identifies Huh-7 and HCC-T cells with the highest potential to study drug metabolism. Arch. Toxicol..

[63] Zhai J.M., Yin X.Y., Lai Y.R., Hou X., Cai J.P., Hao X.Y., Liang L.J., Zhang L.J. (2013). Sorafenib enhances the chemotherapeutic efficacy of S-1 against hepatocellular carcinoma through downregulation of transcription factor E2F-1. Cancer Chemother. Pharmacol..

[64] Hoekstra R., Nibourg G.A.A., Van Der Hoeven T.V., Ackermans M.T., Hakvoort T.B.M., Van Gulik T.M., Lamers W.H., Elferink R.P.O., Chamuleau R.A.F.M. (2011). The HepaRG cell line is suitable for bioartificial liver application. Int. J. Biochem. Cell Biol..

[65] Andersson T.B., Kanebratt K.P., Kenna J.G. (2012). The HepaRG cell line: A unique in vitro tool for understanding drug metabolism and toxicology in human. Expert Opin. Drug Metab. Toxicol..

[66] Xie F., Sun L., Pang Y., Xu G., Jin B., Xu H., Lu X., Xu Y., Du S., Wang Y. (2021). Three-dimensional bio-printing of primary human hepatocellular carcinoma for personalized medicine. Biomaterials.

[67] Sonntag R., Gassler N., Bangen J.M., Trautwein C., Liedtke C. (2014). Pro-apoptotic Sorafenib signaling in murine hepatocytes depends on malignancy and is associated with PUMA expression in vitro and in vivo. Cell Death Dis..

[68] Gu H.R., Park S.C., Choi S.J., Lee J.C., Kim Y.C., Han C.J., Kim J., Yang K.Y., Kim Y.J., Noh G.Y. (2015). Combined treatment with silibinin and either sorafenib or gefitinib enhances their growth-inhibiting effects in hepatocellular carcinoma cells. Clin. Mol. Hepatol..

[69] Mountcastle S.E., Cox S.C., Sammons R.L., Jabbari S., Shelton R.M., Kuehne S.A. (2020). A review of co-culture models to study the oral microenvironment and disease. J. Oral Microbiol..

[70] Miki Y., Ono K., Hata S., Suzuki T., Kumamoto H., Sasano H. (2012). The advantages of co-culture over mono cell culture in simulating in vivo environment. J. Steroid Biochem. Mol. Biol..

[71] Shuichi I., Shimada M., Morine Y., Imura S., Ikemoto T., Saito Y., Yamada S., Rui F. (2019). The effect of hepatic stellate cells on hepatocellular carcinoma progression. J. Clin. Oncol..

[72] Chen W., Wu J., Shi H., Wang Z., Zhang G., Cao Y., Jiang C., Ding Y. (2014). Hepatic stellate cell coculture enables sorafenib resistance in Huh7 cells through HGF/c-Met/Akt and Jak2/Stat3 pathways. Biomed. Res. Int..

[73] Fasolino I., Guarino V., Marrese M., Cirillo V., Vallifuoco M., Tamma M.L., Vassallo V., Bracco A., Calise F., Ambrosio L. (2018). HepG2 and human healthy hepatocyte in vitro culture and co-culture in PCL electrospun platforms. Biomed. Mater..

[74] Lee H.W., Kook Y.M., Lee H.J., Park H., Koh W.G. (2014). A three-dimensional co-culture of HepG2 spheroids and fibroblasts using double-layered fibrous scaffolds incorporated with hydrogel micropatterns. RSC Adv..

[75] Coulouarn C., Corlu A., Glaise D., Guénon I., Thorgeirsson S.S., Clément B. (2012). Hepatocyte-stellate cell cross-talk in the liver engenders a permissive inflammatory microenvironment that drives progression in hepatocellular carcinoma. Cancer Res..

[76] Jung H.R., Kang H.M., Ryu J.W., Kim D.S., Noh K.H., Kim E.S., Lee H.J., Chung K.S., Cho H.S., Kim N.S. (2017). Cell Spheroids with Enhanced Aggressiveness to Mimic Human Liver Cancer in Vitro and in Vivo. Sci. Rep..

[77] Štampar M., Tomc J., Filipič M., Žegura B. (2019). Development of in vitro 3D cell model from hepatocellular carcinoma (HepG2) cell line and its application for genotoxicity testing. Arch. Toxicol..

[78] Takai A., Fako V., Dang H., Forgues M., Yu Z., Budhu A., Wang X.W. (2016). Three-dimensional Organotypic Culture Models of Human Hepatocellular Carcinoma. Sci. Rep..

[79] Wang Y., Takeishi K., Li Z., Cervantes-Alvarez E., de L’Hortet L.C., Guzman-Lepe J., Cui X., Zhu J. (2017). Microenvironment of a tumor-organoid system enhances hepatocellular carcinoma malignancyrelated hallmarks. Organogenesis.

[80] Taymour R., Kilian D., Ahlfeld T., Gelinsky M., Lode A. (2021). 3D bioprinting of hepatocytes: Core–shell structured co-cultures with fibroblasts for enhanced functionality. Sci. Rep..

[81] Sun L., Yang H., Wang Y., Zhang X., Jin B., Xie F., Jin Y., Pang Y., Zhao H., Lu X. (2020). Application of a 3D Bioprinted Hepatocellular Carcinoma Cell Model in Antitumor Drug Research. Front. Oncol..

[82] Ma X., Yu C., Wang P., Xu W., Wan X., Lai C.S.E., Liu J., Koroleva-Maharajh A., Chen S. (2018). Rapid 3D bioprinting of decellularized extracellular matrix with regionally varied mechanical properties and biomimetic microarchitecture. Biomaterials.

[83] Velliou E., Gupta P., Ricci C., Danti S., Subhas C.K., Rui L.R. (2020). Biomaterial-based in vitro models for pancreatic cancer. Materials Today, Biomaterials for 3D Tumor Modeling.

[84] Khanna S., Bhatt A.N., Dwarakanath B.S. (2014). Multicellular Spheroid: 3-D Tissue Culture Model for Cancer Research. Animal Biotechnology.

[85] Fiorini E., Veghini L., Corbo V. (2020). Modeling Cell Communication in Cancer With Organoids: Making the Complex Simple. Front. Cell Dev. Biol..

[86] Rolver M.G., Elingaard-Larsen L.O., Pedersen S.F. (2019). Assessing cell viability and death in 3d spheroid cultures of cancer cells. J. Vis. Exp..

[87] Song Y., Kim J.S., Kim S.H., Park Y.K., Yu E., Kim K.H., Seo E.J., Oh H.B., Lee H.C., Kim K.M. (2018). Patient-derived multicellular tumor spheroids towards optimized treatment for patients with hepatocellular carcinoma. J. Exp. Clin. Cancer Res..

[88] Rijal G., Li W. (2017). A versatile 3D tissue matrix scaffold system for tumor modeling and drug screening. Sci. Adv..

[89] Kapałczyńska M., Kolenda T., Przybyła W., Zajączkowska M., Teresiak A., Filas V., Ibbs M., Bliźniak R., Łuczewski Ł., Lamperska K. (2016). 2D and 3D cell cultures—A comparison of different types of cancer cell cultures. Arch. Med. Sci..

[90] Porter R.J., Murray G.I., McLean M.H. (2020). Current concepts in tumour-derived organoids. Br. J. Cancer.

[91] Costa E.C., Moreira A.F., de Melo-Diogo D., Gaspar V.M., Carvalho M.P., Correia I.J. (2016). 3D tumor spheroids: An overview on the tools and techniques used for their analysis. Biotechnol. Adv..

[92] Fang Y., Eglen R.M. (2017). Three-Dimensional Cell Cultures in Drug Discovery and Development. SLAS Discov..

[93] Kronemberger G.S., Carneiro F.A., Rezende D.F., Baptista L.S. (2021). Spheroids and organoids as humanized 3D scaffold-free engineered tissues for SARS-CoV-2 viral infection and drug screening. Artif. Organs..

[94] Drost J., Clevers H. (2018). Organoids in cancer research. Nat. Rev. Cancer.

[95] Vivarelli S., Candido S., Caruso G., Falzone L., Libra M. (2020). Patient-derived tumor organoids for drug repositioning in cancer care: A promising approach in the era of tailored treatment. Cancers.

[96] Langhans S.A. (2018). Three-dimensional in vitro cell culture models in drug discovery and drug repositioning. Front. Pharmacol..

[97] Chaicharoenaudomrung N., Kunhorm P., Noisa P. (2019). Three-dimensional cell culture systems as an in vitro platform for cancer and stem cell modeling. World J. Stem Cells.

[98] Datta P., Dey M., Ataie Z., Unutmaz D., Ozbolat I.T. (2020). 3D bioprinting for reconstituting the cancer microenvironment. NPJ Precis. Oncol..

[99] Sontheimer-Phelps A., Hassell B.A., Ingber D.E. (2019). Modelling cancer in microfluidic human organs-on-chips. Nat. Rev. Cancer.

[100] Lo Y.H., Karlsson K., Kuo C.J. (2020). Applications of organoids for cancer biology and precision medicine. Nat. Cancer.

[101] Nuciforo S., Heim M.H. (2021). Organoids to model liver disease. JHEP Rep..

[102] Nuciforo S., Fofana I., Matter M.S., Blumer T., Calabrese D., Boldanova T., Piscuoglio S., Wieland S., Ringnalda F., Schwank G. (2018). Organoid Models of Human Liver Cancers Derived from Tumor Needle Biopsies. Cell Rep..

[103] Hoarau-Véchot J., Rafii A., Touboul C., Pasquier J. (2018). Halfway between 2D and animal models: Are 3D cultures the ideal tool to study cancer-microenvironment interactions?. Int. J. Mol. Sci..

[104] Augustine R., Kalva S.N., Ahmad R., Zahid A.A., Hasan S., Nayeem A., McClements L., Hasan A. (2021). 3D Bioprinted cancer models: Revolutionizing personalized cancer therapy. Transl. Oncol..

[105] Bae J., Han S., Park S. (2020). Recent Advances in 3D Bioprinted Tumor Microenvironment. Biochip J..

[106] Wang X., Zhao X., Zhou X., Liu C. (2016). A 3D bioprinting liver tumor model for drug screening. World J. Pharm. Pharm. Sci..

[107] Wu Q., Liu J., Wang X., Feng L., Wu J., Zhu X., Wen W., Gong X. (2020). Organ-on-a-chip: Recent breakthroughs and future prospects. Biomed. Eng. Online.

[108] Trujillo-de Santiago G., Flores-Garza B.G., Tavares-Negrete J.A., Lara-Mayorga I.M., González-Gamboa I., Zhang Y.S., Rojas-Martínez A., Ortiz-López R., Álvarez M.M. (2019). The tumor-on-chip: Recent advances in the development of microfluidic systems to recapitulate the physiology of solid tumors. Materials.

[109] Ehrlich A., Duche D., Ouedraogo G., Nahmias Y. (2019). Challenges and Opportunities in the Design of Liver-on-Chip Microdevices. Annu. Rev. Biomed. Eng..

[110] Lu S., Cuzzucoli F., Jiang J., Liang L.G., Wang Y., Kong M., Zhao X., Cui W., Li J., Wang S.Q. (2018). Development of a biomimetic liver tumor-on-a-chip model based on decellularized liver matrix for toxicity testing. Lab Chip.

[111] Sharifi F., Yesil-Celiktas O., Kazan A., Maharjan S., Saghazadeh S., Firoozbakhsh K., Firoozabadi B., Zhang Y.S. (2020). A hepatocellular carcinoma–bone metastasis-on-a-chip model for studying thymoquinone-loaded anticancer nanoparticles. Bio-Des. Manuf..

[112] Li Z., Zheng W., Wang H., Cheng Y., Fang Y., Wu F., Sun G., Sun G., Lv C., Hui B. (2021). Application of animal models in cancer research: Recent progress and future prospects. Cancer Manag. Res..

[113] Zhang W., Moore L., Ji P. (2007). Mouse models for cancer research. Chinese J. Cancer..

[114] Liu Y., Meyer C., Xu C., Weng H., Hellerbrand C., ten Dijke P., Dooley S. (2013). Animal models of chronic liver diseases. Am. J. Physiol. Gastrointest. Liver Physiol..

[115] Gargiulo G. (2018). Next-generation in vivo modeling of human cancers. Front. Oncol..

[116] Santos N.P., Colaço A.A., Oliveira P.A. (2017). Animal models as a tool in hepatocellular carcinoma research: A Review. Tumor Biol..

[117] Zhang H.E., Henderson J.M., Gorrell M.D. (2019). Animal models for hepatocellular carcinoma. Biochim. Biophys. Acta Mol. Basis Dis..

[118] Guerin M.V., Finisguerra V., Van den Eynde B.J., Bercovici N., Trautmann A. (2020). Preclinical murine tumor models: A structural and functional perspective. Elife.

[119] Maronpot R.R. (2009). Biological basis of differential susceptibility to hepatocarcinogenesis among mouse strains. J. Toxicol. Pathol..

[120] Rogers A.B. (2018). Stress of strains: Inbred mice in liver research. Gene Expr..

[121] Jilkova Z.M., Kurma K., Decaens T. (2019). Animal Models of Hepatocellular Carcinoma: The Role of Immune System. Cancers.

[122] Heindryckx F., Colle I., Van Vlierberghe H. (2009). Experimental mouse models for hepatocellular carcinoma research. Int. J. Exp. Pathol..

[123] He L., Tian D.A., Li P.Y., He X.X. (2015). Mouse models of liver cancer: Progress and recommendations. Oncotarget.

[124] Memon A., Pyao Y., Jung Y., Lee J.I., Lee W.K. (2020). A modified protocol of diethylnitrosamine administration in mice to model hepatocellular carcinoma. Int. J. Mol. Sci..

[125] Da Costa R.M.G., Paula-Santos N., Rocha A.F., Colaç A., Lopes C., Oliveira P.A. (2014). The N-nitrosodiethylamine mouse model: Sketching a timeline of evolution of chemically-induced hepatic lesions. Anticancer Res..

[126] Mohammed E.S., El-Beih N.M., El-Hussieny E.A., EL-Ahwany E., Hassan M., Zoheiry M. (2018). Effects of free and nanoparticulate curcumin on chemically induced liver carcinoma in an animal model. Arch. Med. Sci..

[127] Luo M., Yang F., Huang S.X., Kuang Z.P., Luo X.L., Li Y.D., Wu J.N., Xie Y.A. (2013). Two-stage model of chemically induced hepatocellular carcinoma in mouse. Oncol. Res..

[128] Brown Z.J., Heinrich B., Greten T.F. (2018). Mouse models of hepatocellular carcinoma: An overview and highlights for immunotherapy research. Nat. Rev. Gastroenterol. Hepatol..

[129] Kersten K., Visser K.E., Miltenburg M.H., Jonkers J. (2017). Genetically engineered mouse models in oncology research and cancer medicine. EMBO Mol. Med..

[130] Jung J. (2014). Human tumor xenograft models for preclinical assessment of anticancer drug development. Toxicol. Res..

[131] Qi X., Schepers E., Avella D., Kimchi E.T., Kaifi J.T., Staveley-O’carroll K.F., Li G. (2019). An oncogenic hepatocyte-induced orthotopic mouse model of hepatocellular cancer arising in the setting of hepatic inflammation and fibrosis. J. Vis. Exp..

[132] Chen Q., Wang J., Liu W.N., Zhao Y. (2019). Cancer Immunotherapies and Humanized Mouse Drug Testing Platforms. Transl. Oncol..

[133] Bresnahan E., Lindblad K.E., de Galarreta M.R., Lujambio A. (2020). Mouse Models of Oncoimmunology in Hepatocellular Carcinoma. Clin. Cancer Res..

[134] Reiberger T., Chen Y., Ramjiawan R.R., Hato T., Fan C., Samuel R., Roberge S., Huang P., Lauwers G.Y., Zhu A.X. (2015). An orthotopic mouse model of hepatocellular carcinoma with underlying liver cirrhosis. Nat. Protoc..

[135] Walrath J.C., Hawes J.J., Van Dyke T., Reilly K.M. (2010). Genetically engineered mouse models in cancer research. Adv. Cancer Res..

[136] Singh M., Murriel C.L., Johnson L. (2012). Genetically engineered mouse models: Closing the gap between preclinical data and trial outcomes. Cancer Res..

[137] Ju H.L., Han K.H., Lee J.D., Ro S.W. (2016). Transgenic mouse models generated by hydrodynamic transfection for genetic studies of liver cancer and preclinical testing of anti-cancer therapy. Int. J. Cancer.

[138] Martin J., Dufour J.F. (2008). Tumor suppressor and hepatocellular carcinoma. World J. Gastroenterol..

[139] Chung S.I., Moon H., Kim D.Y., Cho K.J., Ju H.L., Kim D.Y., Ahn S.H., Han K.H., Ro S.W. (2016). Development of a transgenic mouse model of hepatocellular carcinoma with a liver fibrosis background. BMC Gastroenterol..

[140] Guil-Luna S., Sedlik C., Piaggio E. (2020). Humanized Mouse Models to Evaluate Cancer Immunotherapeutics. Annu. Rev. Cancer Biol..

[141] Tian H., Lyu Y., Yang Y.G., Hu Z. (2020). Humanized Rodent Models for Cancer Research. Front. Oncol..

[142] Akkina R. (2013). New generation humanized mice for virus research: Comparative aspects and future prospects. Virology.

[143] Yin L., Wang X., Chen D., Liu X., Wang X. (2020). Humanized mouse model: A review on preclinical applications for cancer immunotherapy. Am. J. Cancer Res..

[144] Maurice Morillon Y., Sabzevari A., Schlom J., Greiner J.W. (2020). The development of next-generation PBMC humanized mice for preclinical investigation of cancer immunotherapeutic agents. Anticancer Res..

[145] Zhao Y., Shuen T.W.H., Toh T.B., Chan X.Y., Liu M., Tan S.Y., Fan Y., Yang H., Lyer S.G., Bonney G.K. (2018). Development of a new patient-derived xenograft humanised mouse model to study human-specific tumour microenvironment and immunotherapy. Gut.

[146] Bi Y., Shi J., Li S., Wang Q., Wang Q., Wen X., Yang F., Duan Z., Yang Y., Zhang X. A novel xenograft model of human HCC in immunocompetent mouse. bioRxiv.

[147] Trisilowati, Mallet D.G. (2012). In Silico Experimental Modeling of Cancer Treatment. ISRN Oncol..

[148] Thoolen B., ten Kate F.J.W., van Diest P.J., Malarkey D.E., Elmore S.A., Maronpot R.R. (2012). Comparative histomorphological review of rat and human hepatocellular proliferative lesions. J. Toxicol. Pathol..

[149] Shankaraiah R.C., Gramantieri L., Fornari F., Sabbioni S., Callegari E., Negrini M. (2019). Animal models of hepatocellular carcinoma prevention. Cancers.

[150] Chen M., Lu S., Zheng H., Xu M., Song J., Yang W., Weng Q., Zheng L., Fan X., Cheng X. (2019). Identification of the Potential Metabolic Pathways Involved in the Hepatic Tumorigenesis of Rat Diethylnitrosamine-Induced Hepatocellular Carcinoma via 1 H NMR-Based Metabolomic Analysis. Biomed. Res. Int..

[151] Ciccarelli O., Colson A., De Saeger C., Reding R., Sempoux C., Leclercq I.A., Stärkel P. (2018). Tumoral response and tumoral phenotypic changes in a rat model of diethylnitrosamine-induced hepatocellular carcinoma after salirasib and sorafenib administration. Onco. Targets. Ther..

[152] Li Y.T., Liu C.J., Su T.H., Cheng H.R., Jeng Y.M., Lin H.L., Wang C.C., Kao J.H., Chen P.J., Chen D.S. (2016). Characterization of metastatic tumor antigen 1 and its interaction with hepatitis B virus X protein in NF-κB signaling and tumor progression in a woodchuck hepatocellular carcinoma model. Oncotarget.

[153] Kim A.Y., Yacoub J.H., Field D.H., Park B.U., Kallakury B., Korolowicz K.E., Menne S. (2020). Suitability of the woodchuck HCC as a preclinical model for evaluation of intra-arterial therapies. Anim. Model. Exp. Med..

[154] Liu L.Y., Ma X.Z., Ouyang B., Ings D.P., Marwah S., Liu J., Chen A.Y., Gupta R., Manuel J., Chen X.C. (2020). Nanoparticle Uptake in a Spontaneous and Immunocompetent Woodchuck Liver Cancer Model. ACS Nano.

[155] Press Z.W. (2021). Application of the woodchuck animal model for the treatment of hepatitis B virus-induced liver cancer. World J. Gastrointest Oncol..

[156] Blair R. (2017). Transarterial Chemoembolization in a Woodchuck Model of Hepatocellular Carcinoma William. Physiol. Behav..

[157] Zhao S., Huang J., Ye J. (2015). A fresh look at zebrafish from the perspective of cancer research. J. Exp. Clin. Cancer Res..

[158] Hason M., Bartůnĕk P. (2019). Zebrafish models of cancer-new insights on modeling human cancer in a non-mammalian vertebrate. Genes.

[159] Huiting L., Laroche F., Feng H. (2015). The Zebrafish as a Tool to Cancer Drug Discovery Current Challenges in Drug Discovery HHS Public Access. Austin J. Pharmacol. Ther..

[160] Zhang T., Peterson R.T. (2019). Zebrafish as a Platform for Drug Screening.

[161] Cassar S., Adatto I., Freeman J.L., Gamse J.T., Lawrence C., Muriana A., Peterson R.T., Van Cruchten S., Zon L.I. (2020). Use of Zebrafish in Drug Discovery Toxicology. Chem. Res. Toxicol..

[162] Xiao J., Glasgow E., Agarwal S. (2020). Zebrafish Xenografts for Drug Discovery and Personalized Medicine. Trends Cancer.

[163] Wrighton P.J., Oderberg I.M., Goessling W. (2019). There Is Something Fishy About Liver Cancer: Zebrafish Models of Hepatocellular Carcinoma. CMGH.

[164] Nakayama J., Gong Z. (2020). Transgenic zebrafish for modeling hepatocellular carcinoma. MedComm.

[165] Nguyen A.T., Emelyanov A., Koh C.H.V., Spitsbergen J.M., Parinov S., Gong Z. (2012). An inducible kras V12 transgenic zebrafish model for liver tumorigenesis and chemical drug screening. DMM Dis. Model. Mech..

[166] Jeanquartier F., Jean-Quartier C., Kotlyar M., Tokar T., Hauschild A.C., Jurisica I., Holzinger A., Holzinger A. (2016). Machine learning for In Silico modeling of tumor growth. Machine Learning for Health Informatics.

[167] Sacan A., Ekins S., Kortagere S. (2012). Applications and limitations of in silico models in drug discovery. Methods Mol. Biol..

[168] Jeanquartier F., Jean-Quartier C., Cemernek D., Holzinger A. (2016). In silico modeling for tumor growth visualization. BMC Syst. Biol..

[169] Jean-Quartier C., Jeanquartier F., Jurisica I., Holzinger A. (2018). In silico cancer research towards 3R. BMC Cancer.

[170] Liu T.P., Hong Y.H., Yang P.M. (2017). In silico and in vitro identification of inhibitory activities of sorafenib on histone deacetylases in hepatocellular carcinoma cells. Oncotarget.

[171] Shi X.N., Li H., Yao H., Liu X., Li L., Leung K.S., Kung H., Lu D., Wong M.H., Lin M.C.M. (2015). In Silico Identification and In Vitro and In Vivo Validation of Anti-Psychotic Drug Fluspirilene as a Potential CDK2 Inhibitor and a Candidate Anti-Cancer Drug Xi-Nan. PLoS ONE.

[172] Sun Y., Zhang Z. (2020). In Silico Identification of Crucial Genes and Specific Pathways in Hepatocellular Cancer. Genet. Test. Mol. Biomark..

[173] Mabrouk M.S. (2012). Discovering best candidates for Hepatocellular Carcinoma (HCC) by in-silico techniques and tools. Int. J. Bioinform. Res. Appl..

[174] Huang S., Yang J., Fong S., Zhao Q. (2020). Artificial intelligence in cancer diagnosis and prognosis: Opportunities and challenges. Cancer Lett..

[175] Azuaje F. (2019). Artificial intelligence for precision oncology: Beyond patient stratification. NPJ Precis. Oncol..

[176] Nagy M., Radakovich N., Nazha A. (2020). Machine Learning in Oncology: What Should Clinicians Know?. JCO Clin. Cancer Inform..

[177] Iqbal M.J., Javed Z., Sadia H., Qureshi I.A., Irshad A., Ahmed R., Malik K., Raza S., Abbas A., Pezzani R. (2021). Clinical applications of artificial intelligence and machine learning in cancer diagnosis: Looking into the future. Cancer Cell Int..

[178] Ho D. (2020). Artificial intelligence in cancer therapy. Science.

[179] Lai Q., Spoletini G., Mennini G., Laureiro Z.L., Tsilimigras D.I., Pawlik T.M., Rossi M. (2020). Prognostic role of artificial intelligence among patients with hepatocellular cancer: A systematic review. World J. Gastroenterol..

[180] Linton-Reid K., John W.C., Belle T. (2020). Introduction: An Overview of AI in Oncology Drug Discovery and Development, Artificial Intelligence in Oncology Drug Discovery and Development.

[181] Kourou K., Exarchos T.P., Exarchos K.P., Karamouzis M.V., Fotiadis D.I. (2015). Machine learning applications in cancer prognosis and prediction. Comput. Struct. Biotechnol. J..

[182] Rashidi H.H., Tran N.K., Betts E.V., Howell L.P., Green R. (2019). Artificial Intelligence and Machine Learning in Pathology: The Present Landscape of Supervised Methods. Acad. Pathol..

[183] Roemer E.J., West K.L., Northrup J.B., Iverson J.M. (2016). Supervised Machine Learning in Oncology: A Clinician’s Guide. Physiol. Behav..

[184] Zou Z.M., Chang D.H., Liu H., Xiao Y.D. (2021). Current updates in machine learning in the prediction of therapeutic outcome of hepatocellular carcinoma: What should we know?. Insights Imaging.

[185] Chaudhary K., Poirion O.B., Lu L., Garmire L.X. (2018). Deep Learning based multi-omics integration robustly predicts survival in liver cancer. Clin. Cancer Res..

[186] Pérez M.J., Grande R.G. (2020). Application of artificial intelligence in the diagnosis and treatment of hepatocellular carcinoma: A review. World J. Gastroenterol..

[187] Sato M., Morimoto K., Kajihara S., Tateishi R., Shiina S., Koike K., Yatomi Y. (2019). Machine-learning Approach for the Development of a Novel Predictive Model for the Diagnosis of Hepatocellular Carcinoma. Sci. Rep..

[188] Abajian A., Murali N., Savic L.J., Laage- F.M., Nezami N., Duncan J.S., Schlachter T., Lin M., Geschwind J., Chapiro J. (2018). Predicting Treatment Response to Intra-arterial Therapies of Hepatocellular Carcinoma using Supervised Machine Learning—An Artificial Intelligence Concept. J. Vasc. Interv. Radiol..

[189] Morshid A., Elsayes K.M., Khalaf A.M., Elmohr M.M., Yu J., Kaseb A.O., Hassan M., Mahvash A., Wang Z., Hazle J.D. (2019). A Machine Learning Model to Predict Hepatocellular Carcinoma Response to Transcatheter Arterial Chemoembolization. Radiol. Artif. Intell..

[190] Huang Y., Chen H., Zeng Y., Liu Z., Ma H., Liu J. (2021). Development and Validation of a Machine Learning Prognostic Model for Hepatocellular Carcinoma Recurrence After Surgical Resection. Front. Oncol..

[191] Saito A., Toyoda H., Kobayashi M., Koiwa Y., Fujii H., Fujita K., Maeda A., Kaneoka Y., Hazama S., Nagano H. (2021). Prediction of early recurrence of hepatocellular carcinoma after resection using digital pathology images assessed by machine learning. Mod. Pathol..

[192] Yamashita T., Kaneko S. (2021). Liver cancer stem cells: Recent progress in basic and clinical research. Regen. Ther..

